# Enhanced power management in PV-Integrated hybrid energy storage systems using fuzzy 2DOF-PI control optimized by hippopotamus algorithm

**DOI:** 10.1038/s41598-026-40106-4

**Published:** 2026-03-16

**Authors:** Hossam Kotb, Ahmed G. Khairalla, Hesham B. ElRefaie, Kareem M. AboRas

**Affiliations:** Department of Electrical Engineering, Faculty of Engineering, Alexandria University, Alexandria, 21544 Egypt

**Keywords:** Photovoltaic systems, Hybrid energy storage, Fuzzy logic control, 2DOF-PI controller, Hippopotamus optimization, Battery–supercapacitor management, DC microgrid, Power management system, Renewable energy integration, Energy science and technology, Engineering, Mathematics and computing

## Abstract

This study presents an advanced control strategy for a standalone photovoltaic (PV) system integrated with a hybrid energy storage system (HESS) comprising batteries and supercapacitors (SCs). The proposed system employs a novel Fuzzy Logic-based Two-Degree-of-Freedom Proportional-Integral (Fuzzy 2DOF-PI) controller, optimized using the Hippopotamus Optimization (HO) algorithm, to enhance power management and stability. The batteries address long-term energy demands, while SCs handle instantaneous power fluctuations, mitigating stress on the batteries and extending their lifespan. The control strategy ensures optimal power distribution, maintains DC bus voltage stability, and prevents battery overcharging by regulating the State of Charge (SOC) within safe limits. The system’s performance is validated through MATLAB/Simulink simulations under varying solar irradiance and load conditions. Comparative analyses with classical PI, Fuzzy PI-based Teaching-Learning-Based Optimization (TLBO), and Particle Swarm Optimization (PSO) demonstrate the better dynamic response, reduced transient time, and minimized overshoot of the proposed approach. Results indicate improvements of at least 15% in peak overshoot and 10% in transient duration, highlighting the robustness and efficiency of the Fuzzy 2DOF-PI controller in hybrid energy storage applications.

## Introduction

### Study background

Recently, there has been an increasing focus on integrating renewable energy sources (RESs) into power generation systems to move towards a more sustainable and environmentally friendly energy mix. This worldwide transition is propelled by the pressing necessity to alleviate climate change, diminish greenhouse gas emissions, and strengthen energy security^1^. Governments and organizations around the world have enacted regulations and offered incentives to accelerate the adoption of RESs, including solar photovoltaic, wind, hydropower, and biomass. These programs have markedly augmented the integration of RESs into power networks, resulting in diversification of energy sources, improved grid resilience, and economic prospects for stakeholders^2^. The inherent variability and fluctuations of RESs pose significant problems for grid stability and energy management^3^. As a result, innovations in energy storage technologies, smart grid infrastructure, and energy management systems have become essential solutions to address these challenges and ensure reliable power supplies^4^. One viable strategy for the effective integration of RESs into power grids is the construction of DC microgrids^5^. DC microgrids have attracted heightened interest owing to their efficiency, reliability, and many uses, such as electric vehicles (EVs), uninterruptible power supplies, and distributed power systems. Unlike traditional AC systems, DC microgrids provide enhanced power conversion efficiency, reduced transmission losses, and streamlined integration with RES and energy storage systems (ESSs). These benefits make DC microgrids a compelling option for improving the sustainability and stability of modern energy infrastructure^6^.

A fundamental component of DC microgrids is the incorporation of hybrid ESSs, which combines multiple storage technologies to improve performance. ESSs can be implemented using several storage technologies, including batteries, supercapacitors, flywheels, and ultracapacitors^7^. Batteries are the most common because of their considerable energy capacity and ability to store large amounts of energy for longer periods. However, sole reliance on batteries in an ESS may lead to reduced battery lifespan and performance degradation, particularly in environments with variable power demands. This limitation arises from the relatively slow response time of batteries to rapid power fluctuations, which may result in increased stress and thermal degradation^8^. To address these challenges, hybrid ESSs integrate multiple storage devices with complementary characteristics, hence enhancing overall system efficiency and reliability^9^. A common HESS configuration involves a combination of batteries and supercapacitors. In this arrangement, supercapacitors, noted for their high-power density and rapid response capabilities, regulate short-term power fluctuations and transient loads. Simultaneously, batteries, noted for their high energy density, provide sustained power over lengthy durations. This synergistic relationship reduces battery strain, extends their lifespan, and improves the overall efficiency of the ESS^10^.

Advanced optimization-based control and planning strategies play a critical role in enhancing voltage regulation and power quality in renewable-integrated distribution systems. Their two-stage reactive power optimization approach demonstrates how coordinated control actions can effectively mitigate voltage deviations and reduce system losses under varying operating conditions. In a related work, the authors extended this concept to a multi-objective, multi-period framework, highlighting the importance of time-varying optimization in accommodating renewable intermittency and load dynamics^11,12^. As well, advanced energy management systems (EMS) are necessary for integrating RESs and energy storage technologies into DC microgrids to maximize energy flow and preserve system stability. EMS are crucial for optimizing operations via real-time monitoring, demand-side management, and adaptive control strategies. Recent EMS solutions incorporate smart grid technology, artificial intelligence (AI), and predictive machine learning techniques to forecast energy consumption, enhance storage efficiency, and bolster grid reliability^13^. Furthermore, the EMS facilitates seamless coordination across RESs, storage devices, and grid infrastructure, mitigating power fluctuations and improving energy efficiency. Despite the numerous advantages of DC microgrids and HESS, certain challenges remain in their widespread implementation. The fluctuation of RESs necessitates suitable control systems to equilibrate supply and demand. Research concentrates on enhancing power interface technology, dynamic energy distribution strategies, and adaptive control methods to improve the reliability of DC microgrids^14^. HESS consisting of batteries and Supercapacitors (SC) may exhibit various topologies, including passive, semi-active, and active configurations^15^. Active topologies have enhanced controllability that allows the full utilization of the storage capacity and power dispatch capabilities of the HESS devices. Each element of the HESS is independently connected to the system bus through a power electronic converter and has a separate control system^16^. Recent studies have demonstrated that metaheuristic optimization-based MPPT algorithms can significantly enhance power extraction, dynamic response, and system stability compared to conventional methods. In particular, advanced bio-inspired optimizers, including Ali Baba and Forty Thieves Optimization (ABFTO) and the Hippopotamus Algorithm (HA), have shown better capability in tracking the global maximum power point under complex operating conditions such as partial shading and rapid irradiance or temperature variations. These intelligent techniques ensure stable power delivery, fast convergence, and effective bidirectional energy management, thereby improving the resilience, efficiency, and sustainability of PV-integrated DC microgrids and EV charging systems^17,18^. The study^19^presents a novel metaheuristic-based control framework that integrates a two-degree-of-freedom PID acceleration (2DOF-PIDA) controller with the recently developed Starfish Optimization Algorithm (SFOA) for temperature regulation of the CSTH process. The 2DOF-PIDA structure improves control performance by independently addressing setpoint tracking and disturbance rejection, whereas the SFOA effectively optimizes the controller parameters through its balanced exploration and exploitation mechanisms. Simulation results confirm the superiority of the proposed approach in terms of tracking precision, disturbance attenuation, and robustness when compared to conventional control techniques^20^.

Advanced studies have demonstrated the effectiveness of learning-based frameworks across load forecasting and battery state estimation. Specifically, in^21^, a spectral attention–enhanced bidirectional memory network showed superior performance in short-term load forecasting by capturing both temporal and spectral features of power demand signals. Meanwhile, the EBWO–GRU–ACKF framework presented in^22^highlighted the integration of optimization algorithms with recurrent neural networks for accurate state-of-charge (SOC) estimation. A multi-task learning (MTL) framework was created in this study to enhance SOH assessment of lithium-ion batteries (LIBs). The framework successfully captures both domain-invariant and target-specific features by using health-dependent pseudo-labels (PLs) and a multi-task strategy, which improves the model’s robustness and generalization abilities^23,24^. Following the same trend, hybrid machine learning methods combining Random Forest, Soft Weight K-Nearest Neighbors, and Levenberg–Marquardt Backpropagation within a variance–covariance weighted framework have been proposed for adaptive parameter tuning. As reported in^25^, incorporating meteorological and temporal variables in these hybrid models reduces errors by 8%–38% and improves forecasting accuracy by 12%–24% compared to single models.

### Literature review

**Fig. 1:**
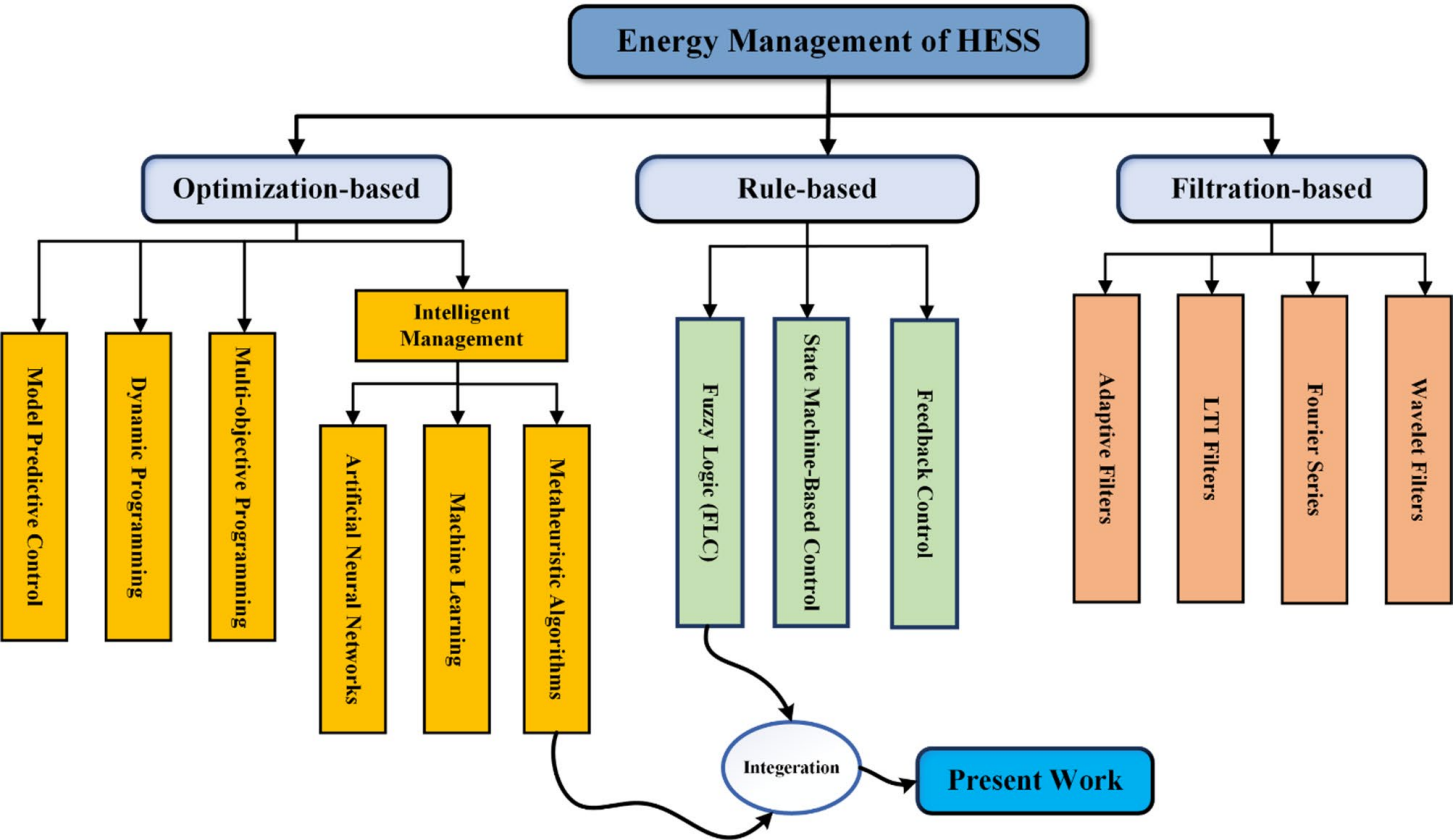
HEES Control Strategies.

Researchers have developed various methodologies for using combined energy sources to send power from a battery and supercapacitor (SC) to the load^26^. Three main approaches exist for HESSs to control their power flow: optimization, filtering, and rule-based models as exhibited in Fig. 1^27^.

The sophisticated techniques encompass data-driven methodologies, including machine learning, artificial neural networks (ANN), and evolutionary algorithms^28^. Following this trend, in Ref^29^., an energy management system utilizing a combination of dynamic programming and neural networks is presented for the HESS, demonstrating near-optimal performance. Nevertheless, the neural network model requires a substantial quantity of sample data for training. Ref^30^. formulated a mathematical model to optimize a hybrid system employing a genetic algorithm (GA). The findings indicate that GA necessitates less time for simulation and demonstrates greater accuracy in delivering outcomes. A notable deficiency of HOMER software is its limited flexibility in model creation. This study analyzed two systems with varying turbine sizes, revealing that turbine size has minimal impact on the outcomes. Authors of^31^employed a multi-objective algorithm to ascertain the dimensions of a HESS in Tanzania. Their findings indicated that incorporating the electrochemical storage system into the HESS enhances its economic viability, particularly in configurations characterized by poor cyclability and shallow depth of discharge.

Recent advancements show that combining hybrid deep learning architectures with metaheuristic optimization significantly enhances temperature prediction accuracy in power system components, thereby improving thermal monitoring and strengthening operational safety and reliability^32^. In addition, accurate wind speed forecasting remains crucial for renewable energy integration, where optimized machine learning frameworks enhance prediction robustness and support stable smart grid operation under varying environmental conditions^33^. To reduce the standardized cost of energy and the corresponding carbon dioxide (CO_2_) emissions that occur throughout the life cycle of the energy system, Ref^34^. used a multi-objective function. For this purpose, they used the Strength Pareto Evolutionary Algorithm. According to the results, photovoltaic (PV) generators have the potential to be a major electrical energy source in Spain. To maximize the size of a hybrid system that combines solar and wind power, Ref^35^. used the Linear TORSCHE optimization technique. According to the results, the cost-effectiveness of the wind, solar, and battery systems together was higher than that of any of the individual systems. This work introduces a hybrid optimization approach, termed DE–HHO, which integrates Differential Evolution (DE) with Harris Hawks Optimization (HHO) to address microgrid scheduling problems under a multi-objective optimization framework that simultaneously minimizes operating costs and environmental impacts. Simulation studies involving wind, photovoltaic, micro-gas turbine, and battery system models demonstrate the superior convergence behavior and global search capability of the proposed DE–HHO algorithm^36^. Moreover, an enhanced Snow Ablation Optimizer incorporating adaptive T-distribution control and Cauchy mutation has been reported to effectively mitigate premature convergence and accelerate convergence speed, highlighting its potential applicability to complex microgrid optimization and energy management problems^37^.

A novel controller FOPI-PI with self-adaptive bonobo algorithm (SABO) and Puma Algorithm (PO) is presented in^38,39^with HESS to reduce the stress on the batteries with load and temperature variations. For a HESS consisting of wind power, photovoltaics, fuel cells, and batteries^40,41^, presented a multi-objective optimization framework using an elephant herding optimization algorithm. To reduce capital costs and improve electrical efficiency and power supply reliability, the proposed approach was studied. The results showed that the recommended approach is suitable for solar photovoltaic system design. The study^42^presents a multi-objective optimization model for microgrid energy management incorporating degradation costs and a carbon trading mechanism to reduce emissions. A hybrid energy storage system smooths renewable fluctuations, while demand response optimizes load. Two novel algorithms, an artificial hummingbird optimizer and a coati optimizer enhanced with advanced ranking and archiving techniques, are proposed to solve the optimization problem. Tested on benchmark functions and IEEE test systems, the coati algorithm improved network loss, voltage deviation, and minimum voltage by up to 56%. Optimal strategies are selected via TOPSIS, demonstrating the model’s effectiveness in managing active distribution networks with renewable integration^43^.

In most microgrid applications, the power management of hybrid energy storage systems is conducted using filtration-based techniques^44^. The established protocol for implementing these techniques involves dividing the current input of the HESS into high-frequency (HF) and low-frequency (LF) components. Subsequently, the HF components get designated for the SC. While using linear time invariant (LTI) low-pass filters (LPF) for power smoothing reduces system complexity, efficiency is sacrificed in the process. On the other hand, sophisticated filtering methods like wavelet transformations can be used to improve system efficiency, but doing so comes at the cost of the charge control system’s computing complexity^45,46^. Using less-than-ideal filters in practice could cause the supercapacitor to fully charge or discharge. Furthermore, unexpected variations in the HESS’s input power may place a lot of strain on the SC, which has the ability to instantly fully charge or discharge the SC. Adaptive filtering techniques can be used to improve system efficiency and stop state of charge (SOC) violations in SC^47^. A rule-based controller is usually used in adaptive rule-based filters to relax the filter in the event that the SC’s SOC exceeds a predetermined threshold. To avoid SOC violation in this instance, extra HF components of the HESS input power are delivered to the BESS. As a result, the filter’s bandwidth and net power variations should be taken into account while designing the SC’s size. Otherwise, the filter is frequently turned off, which could reduce the effectiveness of the system. Model predictive control (MPC) can regulate the output voltage and current of power converters at the primary control level of microgrids. For instance, a rapid model predictive control (MPC) is proposed in research^48^. This MPC controller increases the robustness of DCMGs against a variety of disturbances by using just local information in the HESS. Simplified switching states and a one-step prediction horizon allow for rapid regulation of the DC bus voltage. Additionally, the residual capacity prompted activating sequence of various ESS types based on a dynamic voltage control optimizes the power allocation command.

Conversely, rule-based approaches exhibit reduced computing complexity and are better appropriate for real-time applications. There are two types of rule-based approaches: fuzzy rule-based systems and finite state machines (FSMs). The rules in these approaches could be developed by a specialist or taken from mathematical models. Table 1 summarizes the latest techniques of fuzzy logic control (FLC) in HESS.

**Table 1:** Summary of FLC methodologies utilized in HESS.

Ref.	Control Strategy	RESs	ESDs	Main Features	Significant Shortcomings
^49^	FLC to manage DC/DC converters. A rule-based overhead controller determines operating modes.	-	Li-ion batteries + Ultracapacitor Bank (UC)	Implements seven different operating modes depending on load demand and SoC of energy storage devices. Uses two DC/DC converters (boost and buck) and a switch for direct battery-load connection.	Elevated complexity due to the suggested approach necessitating several converters, sensors, switches, and a microcontroller-based control system, despite the absence of a RES. High overshoot in battery and UC responses as Step changes in buck converter duty cycle cause transient voltage overshoot.
^50^	FLC integrated with the harmony search algorithm (HSA) is used to optimize the hybrid energy system.	Photovoltaic (PV) + Wind turbines	Battery bank + Diesel Generator (DG): backup	Sensitivity analysis concerning the probability of loss of power supply (LPSP) has been examined across several scenarios. Determines optimal sizing of PV, wind, battery, and DG to minimize costs and emissions EMS prioritizes RES (solar/wind), then storage (batteries), and lastly the diesel generator.	While the fuzzy HSA shows promising results, the paper does not extensively compare it with other optimization algorithms. A more comprehensive comparison could provide a clearer understanding of its relative advantages and limitations. The study does not consider load-shifting techniques or demand-side management to balance energy supply and demand.
^51^	FLC combined with ant colony optimization (ACO) to enhance DC microgrid (DC-MG) performance	Photovoltaic (PV)	Battery (Li-ion) + Supercapacitor (SC) + Fuel Cell (FC)	Fuzzy logic control (FLC) manages energy flow and stabilizes voltage. Voltage sag reduction by 98% using FLC in single-line-to-ground faults (SLGF), 97% in dual-line-to-ground faults (DLGF), and 96% improvement in triple-line-to-ground faults (TLGF).	The control strategy does not explicitly address rapid fluctuations in renewable energy sources which represents a highly expected challenge in such area. The system might struggle with sudden load changes or unpredicted disturbances in microgrid operations.
^52^	FL-Based Particle Swarm Optimization (FLB-PSO) algorithm for hybrid energy management.	Solar Photovoltaic (PV)	Battery Energy Storage System (BESS)	The approach improved utilization of BESS, enhancing total system cost and efficiency. The three evaluated battery operational modes show that the system proficiently optimized grid power acquisitions and battery deterioration expenses. The proposed strategy accounts for fixed and dynamic grid tariffs to optimize cost savings.	The proposed methodology employs an outdated optimization technique, in rapidly evolving environments (e.g., real-time energy systems), PSO may not adjust quickly unless enhanced by additional adaptive techniques. The study includes battery deterioration costs but neglects temperature factors and charging rates. The study fails to examine alternate devices such as supercapacitors, or hydrogen storage, which may reduce deterioration problems.
^53^	Uses a Teaching–Learning-Based Optimization (TLBO) algorithm to fine-tune fuzzy logic control for energy management.	-	Li-ion batteries + Ultracapacitor Bank (UC) + EVs	In comparison to conventional (PSO) and non-optimized fuzzy logic, TLBO decreases battery power consumption. Battery power demand decreased by 6.4% relative to PSO-F and 35% compared to NO-F. Comparative analyses are performed on the power-sharing capabilities under two specific driving conditions: the urban dynamometer driving schedule and the European extra-urban driving cycle.	The absence of solar panels or alternative energy harvesters necessitates reliance on grid charging, resulting in elevated operational expenses. The Teaching–Learning-Based Optimization (TLBO) algorithm requires intensive computation, which may not be suitable for real-time embedded controllers in EVs. The paper utilizes static fuzzy logic rules optimized by TLBO but does not investigate adaptive or AI-driven learning methodologies such as: Self-learning metaheuristic algorithms
^54^	Combines PI control FLC to enhance energy management in Fuel Cell Electric Vehicles (FCEVs)	-	Battery (Li-ion) + Supercapacitor (SC) + Fuel Cell (FC)	PI control ensures stable operation, while FLC provides dynamic adaptability. Enhancements in Performance Concerning PI and FLC showing hydrogen usage is 7.53% lower in comparison to PI control and 5.5% elevated State of Charge (SOC) relative to Full Charge (FLC), enhancing battery longevity. Overall efficiency improvement of 3.43% compared to PI and 9.1% compared to FLC.	The study assumes fixed battery and supercapacitor sizes, without addressing scalability for different vehicle types (e.g., heavy-duty EVs) While the study optimizes hydrogen consumption, it does not explicitly model long-term PEMFC degradation. The paper does not include load-shifting strategies, such as: Predictive energy management based on driving behavior and Vehicle-to-grid (V2G) interactions to optimize grid energy use. The system relies solely on hydrogen fuel cells, with no provision for solar or wind energy.
^55^	The proposed control legislation utilizes an optimized fuzzy logic methodology known as the signed-distance or single input FLC.	Solar Photovoltaic (PV)	Battery + Supercapacitor (SC)	The EMS presents numerous advantages compared to traditional control laws, such as the removal of the necessity for an accurate mathematical model in controller design, resilience to parameter fluctuations, and enhanced stability amid variations in production or load. Reduces the number of fuzzy rules, making it simpler than conventional FLC.	The mathematical representations for battery and supercapacitor are very simplified which lead to the relatively inaccurate analysis of battery degradation, temperature impacts, and the aging of storage components. The proposed SDFLC is compared with PI controllers, but not with modern AI-based methods like machine learning-based predictive control, model predictive Control (MPC), and sliding mode control (SMC
^56^	Implements a Fuzzy Logic Supervisor (FLS) for energy management.	Photovoltaic (PV)	Battery (Li-ion) + Supercapacitor (SC)	EMS is engineered to manage power distribution across storage devices by determining the ideal operating mode, therefore guaranteeing a consistent supply to the load while keeping the state-of-charge of SCs and batteries within permissible thresholds.	Fuzzy logic exhibits limited adaptability due to its reliance on predefined rules, which are not capable of self-learning.AI-driven techniques (e.g., Reinforcement Learning, Genetic Algorithms, or Neural Networks) may provide enhanced adaptive and optimal control. Conventional PI controller is utilized and don not compared with other controllers
^57^	The suggested approach integrates the Mud Ring Algorithm (MRA) with a FLC. Thus, it is designated as MRA-FLC.	PV + Wind turbines	Battery Bank + Ultracapacitor Bank (UC) + EVs	The MRA-FLC approach efficiently distributes power between renewable sources, storage systems, and EV chargers. The proposed system achieves 96% efficiency, outperforming other methods such as Particle Swarm Optimization (PSO) (85%), Ant Lion Optimizer (ALO) (93%), and Salp Swarm Algorithm (SSA) (90%).	The discussed cases do not include the variation of load in and RESs power. The paper focuses on efficient power management but does not analyze battery degradation over time. The system is compared to PSO, ALO, and SSA, but not with advanced AI techniques like deep learning or reinforcement learning-based energy management systems.
^58^	Supervisory Fuzzy Logic-Based Energy Management Technique (FL-EMT) for DC microgrids.	PV + Wind turbines	(BESS) + Supercapacitor (SC)	The proposed primary attributes of FLEMS include equitable power distribution among sources, storage devices, and demand loads, optimization of flow rates, discharge, and charge cycles of energy storage devices, and extension of their operational lifespans. The suggested FL-EMT has been achieved the following outcomes: efficient power distribution, rapid adjustment of DC link voltage regarding overshoot and stability, and maintenance of the SoC of the HESS within specified limits.	The FL-EMT control strategy requires complex fuzzy logic rule sets (27 scenarios) which may not be suitable for low-power embedded controllers due to processing demands. The study does not account for dynamic electricity pricing, demand response, or time-of-use tariffs The fuzzy rule set is designed for predefined conditions, but it is not dynamically optimized using any algorithms.
^59^	This work introduces a novel energy management technique for HESS in electric vehicles, optimized by genetic algorithms and fuzzy control.	-	Battery (Li-ion) + Supercapacitor (SC)	The genetic algorithm is employed to optimize the configuration of the fuzzy membership function, aiming to minimize energy loss as the objective. The strategy is validated across different temperatures (10 °C, 25 °C, and 40 °C). The strategy enhances energy efficiency by 2.6% to 3.3%, depending upon temperature variations.	The study does not integrate solar or wind power, which could enhance sustainability. While the approach accounts for temperature, it lacks real-time dynamic adjustment. In comparison to other approaches, GAs may require more time to converge to an ideal solution.
^60^	The research suggests a first order sliding mode control (NBF-FOSMC) for a hybrid hydrogen-electric system (HESS) in a DC microgrid that is based on a nonlinear barrier function. The system utilizes a fuzzy logic-based PMS for optimal power distribution.	Photovoltaic (PV)	Battery Bank + Fuel Cell (FC) + Superconducting Magnetic ES	The controller is divided into two levels: first, master level control which generates power references based on state of charge (SoC) of the energy storage devices and load requirements. Second, slave level control which Regulates the power sources by tracking reference currents. Combination of multiple energy storage types: Hydrogen storage for long-term energy, battery for steady power, and SMES for fast response.	Tuning of the fuzzy logic controller and NBF-FOSMC parameters is complex and mainly depends on expert knowledge. The study does not provide a clear method for selecting optimal control parameters The fuel cell response time is significantly slower than batteries or superconducting magnetic energy storage (SMES). During high transient load demands, the fuel cell may struggle to keep up, requiring additional storage support. The comparison with Lyapunov redesign control only focuses on DC bus voltage stability.

### Paper contribution

This study employs a novel control architecture to guarantee the stability and robustness of interconnected micro-DC grids. The suggested controller parameters can be modified via Hippopotamus Optimization (HO) technique^61^. This study’s unique contributions, in contrast to prior research, are distinctly apparent in the following main aspects:

- Proposing an innovative control method that combines fuzzy logic with 2DOF-PI controller to manage the power of solar panels, batteries, and supercapacitors.
- With sophisticated modeling for both SC and batteries, this study suggests a novel optimized EMS for a battery–SC that is executed in a full-active configuration utilizing dual converters.
- The adoption of a 2DOF-PI control structure, allowing independent tuning of reference tracking and disturbance rejection, which is rarely considered in existing HESS fuzzy–PI designs that typically rely on 1DOF structures.
- The coordinated integration of a fuzzy supervisory layer with the 2DOF-PI controllers governing dual bidirectional converters in a fully active HESS.
- The suggested F2DOF-PI controller employs a HO method to refine its parameters. This novel optimization technique is being implemented for the first time in the domain of micro-DC grids.
- The novel control architecture presents numerous benefits compared to existing controllers by integrating the merits of fuzzy logic with 2DOF-PI. Consequently, enhanced stability, reliable performance, resilience, and better transient response can be attained. Moreover, in contrast to the classical methodology illustrated in^62^, and Fuzzy logic with PI controller based PSO and TLBO illustrated in^52,53^, the suggested controller distinctly surpasses all other controllers in essential aspects, including transient response attributes such as transient time, and overshoot/undershoot.
- The simulation encompasses four different scenarios pertaining to solar radiation and load variance. The outcomings show an improvement in peak overshoots by at least 15% in all cases and 10% in transient duration.

The paper is organized in the following way: Sect. 2 outlines the detailed configuration and modelling of the system. Section 3 outlines the suggested control scheme, the DC bus configuration, the suggested controller, and the proposed optimization technique (HO) and its many strategies. Section 4 elucidates the simulation outcomes, thoroughly examining solar radiation and load variations. Section 5 ultimately delineates the research conclusions and findings.

## Structure and modeling of the proposed system

**Fig. 2:**
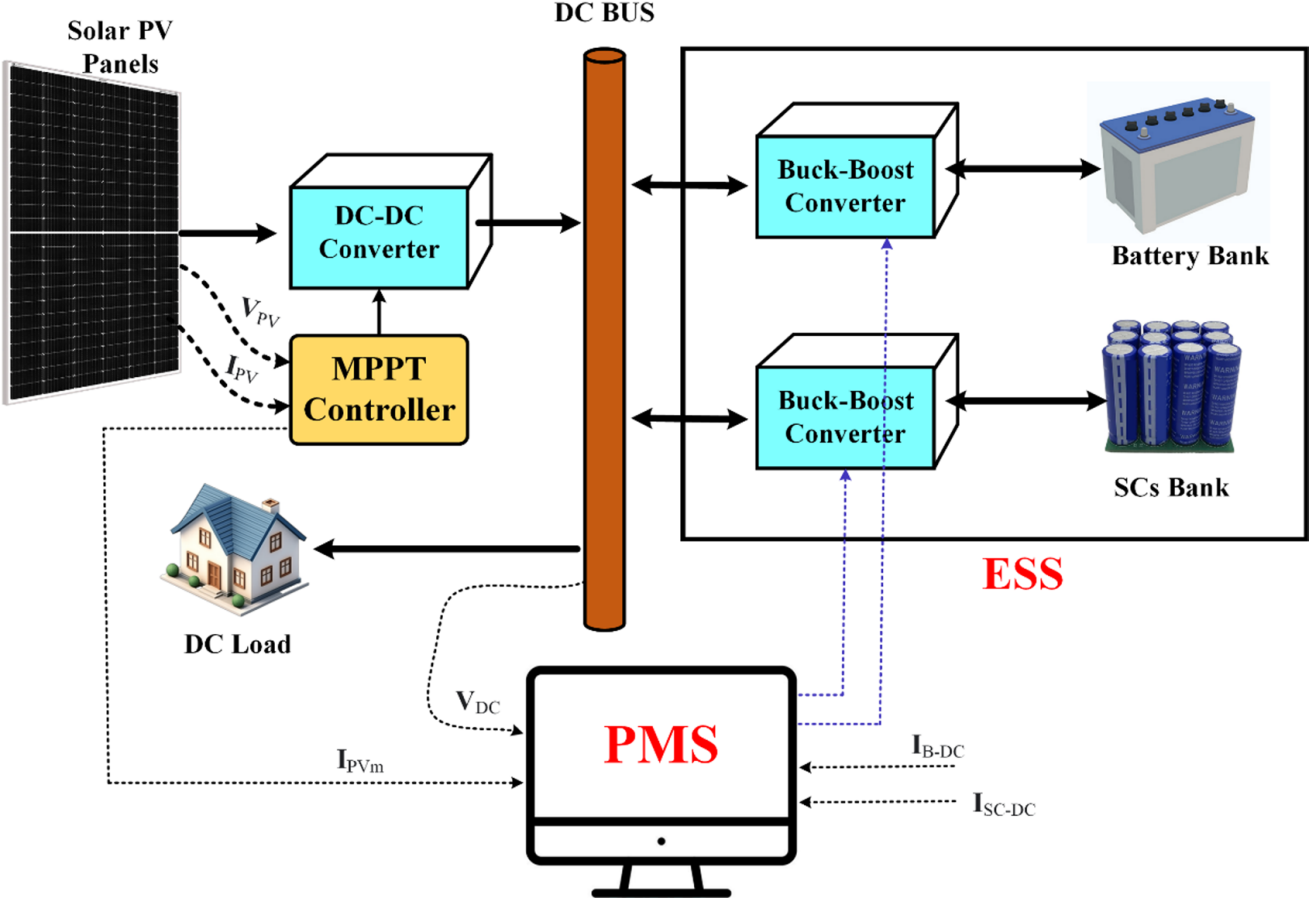
Complete architecture of a HES with PV.

Figure 2 shows a complete design for a solar-powered hybrid energy management system that is meant to make power distribution and storage in DC microgrids more efficient. A MPPT controller controls this power by dynamically changing the operating point to get the most energy out of the PV voltage (ₚ_v_) and current (ₚ_v_). Then, the regulated DC power is sent to a centralized DC bus. There are a lot of parts connected to the DC bus, such as the DC load and an ESS, which is made up of a battery bank and a supercapacitor (SC) bank. Both storage units connect to the DC bus using separate buck-boost converters, which let energy flow in both directions for charging and discharging. The Power Management System (PMS) is in charge of the whole system and makes smart choices to keep the system stable and running at its best by balancing the generation, storage, and use of energy. An active topology’s main benefit is that it actively controls each ESS’s power. Active topologies fall into two categories: parallel and cascaded. A battery and supercapacitor (SC) ESS with a parallel active architecture was suggested in^63^. In microgrids (MG), the parallel active topology is widely adopted due to several key advantages. This configuration offers enhanced flexibility by allowing independent control of HESS units, enabling a wide range of control techniques to be implemented. Moreover, the voltage levels of the Energy Storage System (ESS) units do not directly affect the system voltage, which simplifies system integration and design. Additionally, the parallel active topology improves the system’s inherent fault tolerance, contributing to increased reliability and stability of the microgrid^64^.

### Setup of PV model

**Fig. 3:**
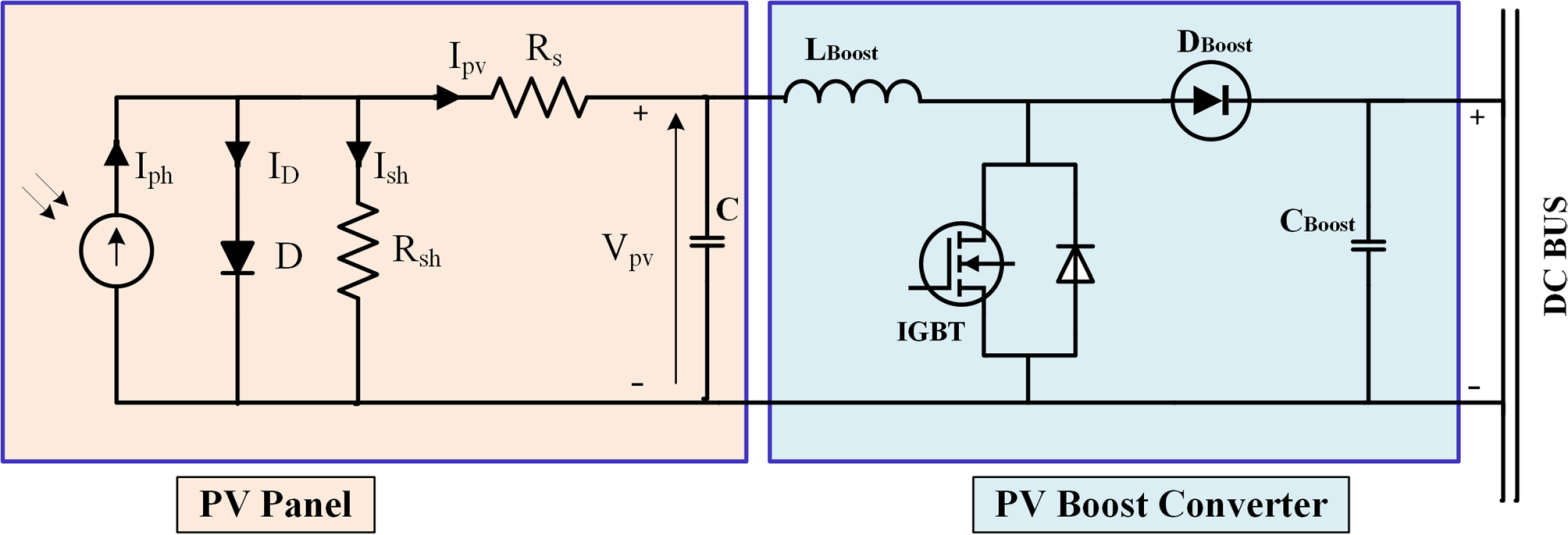
Circuit diagram of PV panel with boost converter.

The constructed model of a photovoltaic cell entails the computation of current-voltage and power-voltage characteristics utilizing exact formulae. Researchers have developed models utilizing one to five factors. The five-parameter approach is the most favored and dependable, particularly in outdoor environments^65^. Figure 3 depicts the execution of the photovoltaic model. The model for a photovoltaic cell comprises many components: I_ph_ denotes the sunlight current, I_D_ signifies the diode current, and Ish represents the shunt-leakage current. Furthermore, I_pv_ denotes the output current supplied by the panel, while R_s_ represents the series resistance^66^. The output current is calculated from a series of equations from (1) to (4):

1 $$\:\mathrm{I}\mathrm{pv}\mathrm{=}\text{}\mathrm{I}\mathrm{ph}\mathrm{\:\--\:I}\mathrm{D}\mathrm{\:\--\:I}\mathrm{sh}\text{}$$

2 $$\:\mathrm{I}\mathrm{pv}\mathrm{\:=\:}\mathrm{I}\mathrm{ph}\mathrm{\:\--\:I}\mathrm{s}\text{}\mathrm{[exp((}\text{}\frac{\mathrm{q(}\mathrm{V}\mathrm{t}\mathrm{+}\mathrm{I}\mathrm{pv}\mathrm{R}\mathrm{s}\mathrm{)}}{\mathrm{kT}\mathrm{c}\mathrm{\:A}}\text{}\mathrm{)}\text{}\mathrm{\--}\mathrm{1)\:}\mathrm{\--}\mathrm{\:(\:}\frac{\mathrm{V}\mathrm{t}\mathrm{+}\mathrm{I}\mathrm{t}\mathrm{R}\mathrm{s}}{\mathrm{R}\mathrm{sh}}\mathrm{\:)]}$$

3 $$\mathrm{I}\mathrm{s}=\mathrm{Ish}=\:I\mathrm{sh}(\frac{\mathrm{T}\mathrm{C}}{\mathrm{T}\mathrm{ref}})\mathrm{3}\times \mathrm{exp}[\mathrm{qG}\mathrm{r}\:(\frac{\mathrm{1}}{\mathrm{T}\mathrm{ref}}-\:\frac{1}{\mathrm{T}\mathrm{C}})\times \frac{1}{\mathrm{kA}}]$$

4 $$\:\mathrm{I}\mathrm{pv}\:=\mathrm{N}\mathrm{p}\mathrm{I}\mathrm{ph}\mathrm{\:\--\:}\mathrm{N}\mathrm{p}\mathrm{I}\mathrm{s}\mathrm{[}\mathrm{exp}\mathrm{((\:}\frac{\mathrm{q\:(\:}\frac{\mathrm{V}\mathrm{t}}{\mathrm{N}\mathrm{s}}\mathrm{\:+\:}\frac{\mathrm{R}\mathrm{s}}{\mathrm{N}\mathrm{p}}\mathrm{\:I}\mathrm{pv}\mathrm{\:)}}{\mathrm{kT}\mathrm{c}\mathrm{\:A}}\mathrm{\:)\:}-\mathrm{1)\:}-\mathrm{\:(\:}\frac{\frac{\mathrm{N}\mathrm{p}}{\mathrm{N}\mathrm{s}}\mathrm{\:V}\mathrm{t\:}\mathrm{+\:}\mathrm{I}\mathrm{pv\:}\mathrm{R}\mathrm{s}}{\mathrm{R}\mathrm{sh}}\mathrm{\:)]}$$

Where N_p_ represents the quantity of solar cells arranged in parallel,, Electron charge (q), cell output voltage (V_PV_), cell output current (I_PV_), number of series-connected cells (N_s_), ideality factor (A), Boltzmann constant (K), and temperature (T) are all variables in this equation. A DC-DC buck-boost converter has been employed for the regulation of the PV array linked to the DC bus, enabling the elevation of voltage from the PV module to sustain the load voltage at the specified level. The solar panel under consideration has a peak power output of 120 W, achieved at a maximum power point (MPP) current of 7.1 A and a voltage of 17 V. Under no-load conditions, the panel exhibits (V_oc_) of 21 V, while (I_sc_) reaches 8 A. The panel’s electrical performance is also influenced by temperature variations, with a short-circuit current temperature coefficient of + 0.052%/°C, indicating a slight increase in current with rising temperature, and an open-circuit voltage temperature coefficient of − 0.358%/°C, reflecting a typical decrease in voltage as temperature increases. These characteristics are essential for accurately modeling the panel’s behavior under varying environmental conditions and optimizing its integration within solar energy systems.

### Setup of SC model

**Fig. 4:**
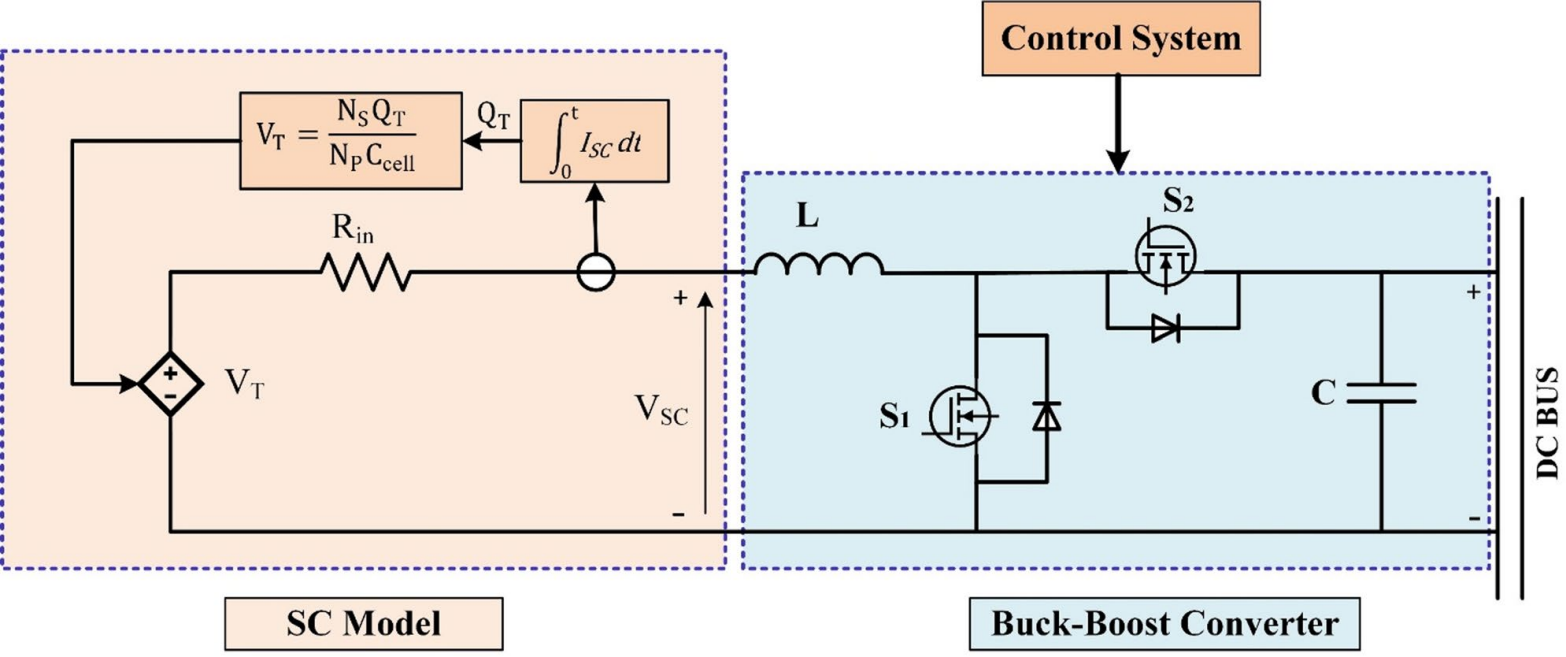
Circuit diagram of SC with buck-boost converter.

The SC operates as an electrical element with a high-power density and a quick dynamic response. The hybrid system may either release excess power or store additional energy from regeneration to make up for the large variation in power consumption. In this study, a SC model is constructed using the Stern model^67^. The SC model’s circuit is shown in Fig. 4. The SC voltage can be expressed as follows:

5 $$\:{\mathrm{V}}_{\mathrm{SC}\text{}}\mathrm{=}\text{}\frac{{\mathrm{N}}_{\mathrm{S}}{\mathrm{Q}}_{\mathrm{T}}}{{\mathrm{N}}_{\mathrm{P}}{\mathrm{C}}_{\mathrm{cell}}}-{\mathrm{R}}_{\mathrm{SC}}{\text{}\mathrm{I}}_{\mathrm{SC}}$$

where I_SC_ is the current flowing through the SC, R_SC_ is the internal resistance, N_S_ and N_P_ are the cells in series and parallel, respectively, and Q_T_ is the total electric charge (in coulombs). The SC energy E_SC_ is determined by two factors: the SC voltage V_SC_ and the SC capacitance Q_SC_^68^:

6 $$\:{\mathrm{E}}_{\mathrm{S}\mathrm{C}}=\frac{{\mathrm{Q}}_{\mathrm{S}\mathrm{C}}\cdot\:{\mathrm{V}}_{\mathrm{S}\mathrm{C}}^{2}}{2}$$

As a result, the quantity of energy stored will fluctuate in proportion to changes in the capacitor’s voltage, and the SOC_SC_ may be computed as follows:

7 $$\:\mathrm{SO}{\mathrm{C}}_{\mathrm{SC}}\mathrm{=}\left(\mathrm{SO}{\mathrm{C}}_{\mathrm{SC}}\left({\mathrm{t}}_{\mathrm{0}}\right)-\frac{\int\:{\mathrm{I}}_{\mathrm{SC}}{\hspace{0.17em}}\mathrm{dt}}{{\mathrm{Q}}_{\mathrm{SC}}}\right)\mathrm{*100\%}$$

SC is linked to the DC bus using a standard buck-boost converter. This converter is made by replacing the unidirectional switches of a normal buck and boost converter with bidirectional power switches. The final product is a BDC that can be used as a buck converter in the opposite direction and as a boost converter from V_sc_ to V_dc_^69^. The parameters of the SC utilized in this model are presented briefly in Table 2.

**Table 2:** Parameters of the SC.

Parameter	Value
Rated capacitance	29 F
Equivalent DC resistance	0.003 Ω
Rated voltage	32 V
Operating temperature	25 °C
Permittivity of electrolyte material	6.0208e-10 F/m

### Setup of battery model

**Fig. 5:**
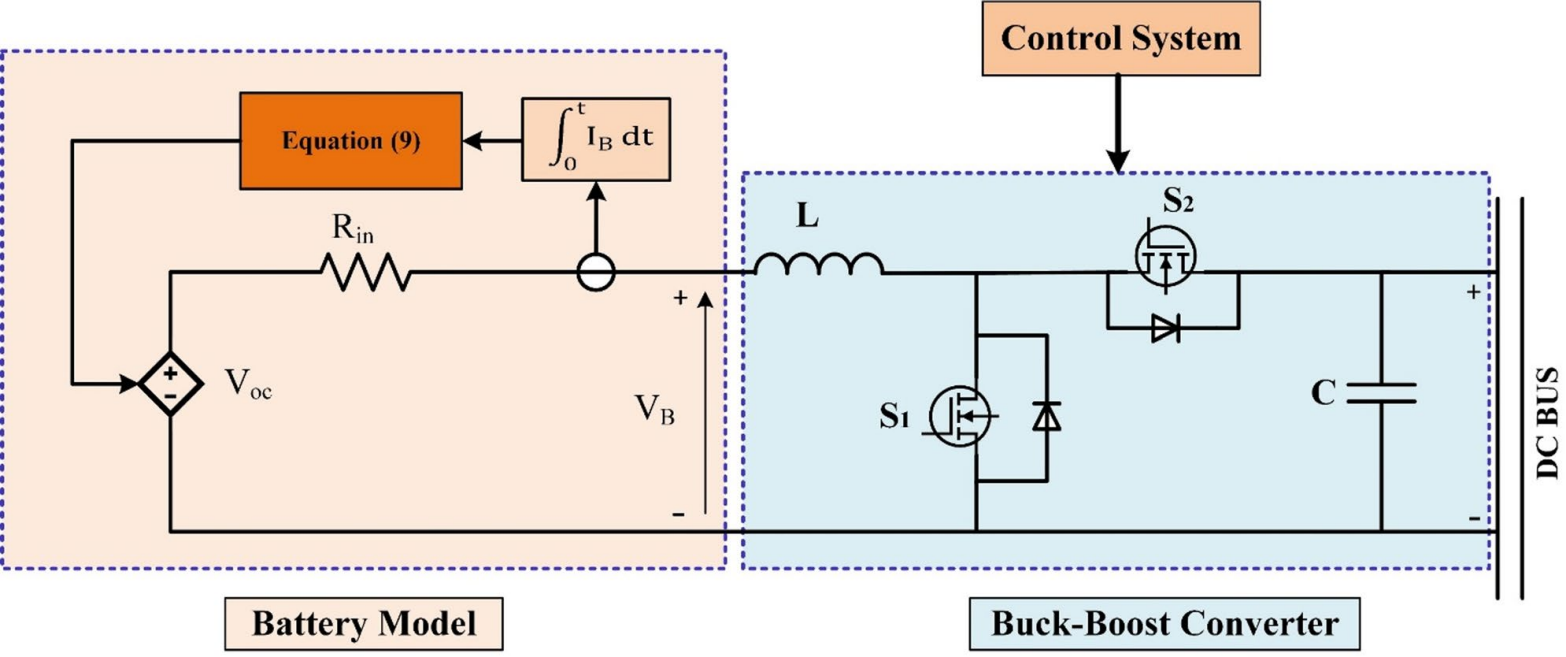
Circuit diagram of battery with buck-boost converter.

ESTs are often governed to monitor the energy exchange between the generating and load sectors under both normal and abnormal circumstances. Furthermore, the role of ESTs becomes crucial, especially when the optimal utilization of renewable energies is implemented. The current work used a typical battery model in which the state of charge (SOC) is treated as a state variable to mitigate arithmetic loop complexity and to enable the representation of four battery varieties, including the lead-acid variant employed in this research^70^. The model characterizes the battery as a regulated voltage source in conjunction with constant resistance, as illustrated in Fig. 5 and highlighted by Eqs. (8) and (9).

8 $$\:\mathrm{V=}{\text{}\mathrm{V}}_{\mathrm{0}}-\frac{{\mathrm{V}}_{\mathrm{Pol}}{\text{}\mathrm{C}}_{\mathrm{bat}}}{{\mathrm{C}}_{\mathrm{bat}}-{\int\:}_{\mathrm{0}}^{\mathrm{t}}{\mathrm{i}}_{\mathrm{B}}{\hspace{0.17em}dt}}\mathrm{+A}\mathrm{exp}\left(-\mathrm{B}{\int\:}_{\mathrm{0}}^{\mathrm{t}}{\mathrm{i}}_{\mathrm{B}}{\hspace{0.17em}dt}\right)$$

9 $$\:{\mathrm{V}}_{\mathrm{B}}\mathrm{=}\text{}\mathrm{E}-{\mathrm{R}}_{\mathrm{in}}\text{}{\mathrm{I}}_{\mathrm{B}}$$

The no-load voltage, constant voltage of the battery, polarization voltage, battery capacity, real battery charge, amplitude of the exponential zone, and inverse of the time constant of the exponential zone are represented by V, V_0_, V_Pol_, C_bat_, ∫i_B_ dt, A, and B, respectively, in the relationships given above. V_B_ denotes the battery voltage, R_in_ represents the internal resistance, and i_B_ indicates the real battery current. The maximum capacity and the change of current charge can be used to identify the battery’s state of charge (SOC).

10 $$\:\mathrm{SOC=100}\left(\mathrm{1+}\frac{\int\:{\mathrm{I}}_{\mathrm{bat}}{\hspace{0.17em}dt}}{{\mathrm{C}}_{\mathrm{bat}}}\right)$$

The parameters of the battery utilized in this model are presented briefly in Table 3.

**Table 3:** Parameters of the Battery.

Parameter	Value
Nominal voltage	24 V
Internal resistance	0.017 Ω
Rated capacity	14 Ah

## Proposed control and management system

### Concept of the proposed control methodology

**Fig. 6:**
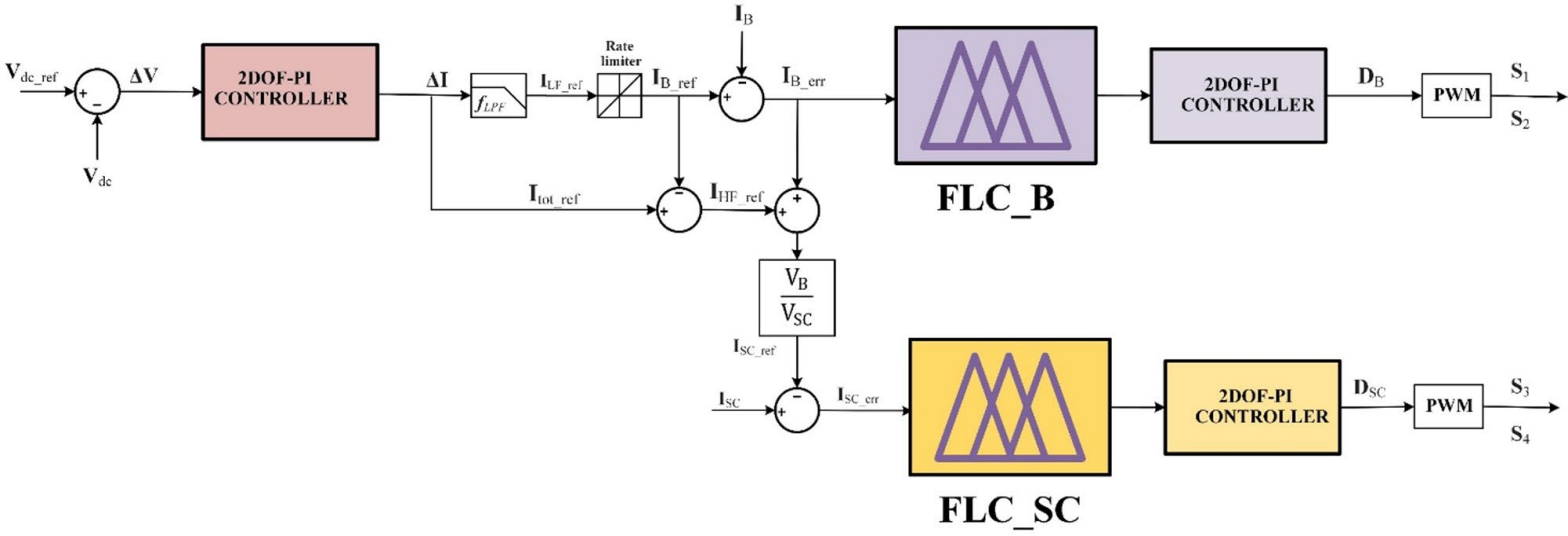
Proposed HESS Control Scheme.

An illustration of the proposed control technique may be found in Fig. 6. With this approach, the goal is to reduce the amount of strain that is placed on batteries throughout the charging and discharging cycles, hence extending the lifespan of the batteries. It is anticipated that the state of charge (SOC) of the batteries would continually remain within a range that is considered to be acceptable. In order for the method to function, it first compares the mean value of Vdc with a reference voltage (Vref), and then it sends the error to a proposed controller. The output signal of the proposed controller is represented by the total current (ΔI). Using Eq. (11), one can get the total current that is required from the HESS, which is comprised of both batteries and supercapacitors (SCs)^62^.

11 $$\:\mathrm{ΔI\:=\:}\mathrm{I}\mathrm{pv}\mathrm{\:\--\:}\mathrm{I}\mathrm{load}\text{}\mathrm{=\:I}\mathrm{B}\mathrm{\:+\:I}\mathrm{S}\mathrm{C}$$

Based on frequency, the reference current I_tot_ref_ is separated into a (I_LF_ref_) and a (I_HF_ref_). The current (I_LF_ref_) is fulfilled by the batteries following the rate-limiting operation, which may be achieved through the use of a low-pass filter. In contrast, the SCs may satisfy the (I_HF_ref_). The LF component can be defined as:

12 $$\:\mathrm{I}\mathrm{LF\_ref}\text{}\text{}\mathrm{=}\text{}\mathrm{f}\mathrm{LPF}\text{}\mathrm{(\:}\mathrm{I}\mathrm{tot\_ref}\text{}\mathrm{)}$$

Where ***f***_LPF_ is the low-pass filter TF.

So, the current of the battery may be:

13 $$\:\mathrm{I}\mathrm{B\_ref}\text{}\mathrm{=}\text{}\mathrm{f}\mathrm{RL}\mathrm{(\:}\mathrm{I}\mathrm{LF\_ref}\mathrm{)}\text{}$$

Where ***f***_RL_ is the rate limiter TF.

In the proposed control framework, the rate limiter applied to the battery reference current in (14) is introduced to account for the inherently slower dynamic characteristics of batteries compared to supercapacitors and to mitigate excessive current stress. As indicated by (12) and (13), the total reference current is first decomposed into low and high-frequency components using a low-pass filter with a time constant of 0.015 s, and the resulting low-frequency component is then processed through a rate-limiting function. This ensures that the battery supplies only the slowly varying component of the load demand, whereas rapid current transients and high-frequency power fluctuations are primarily absorbed by the supercapacitor, thereby alleviating potential current stress on the battery and contributing to reduced degradation. The control method that has been suggested involves comparing the (I_B_ref_) with the actual (I_B_) and then entering the error signal into the fuzzy controller that has been provided. Following that, the 2DOF-PI does the calculation necessary to determine the duty ratio (D_Bat_) that is generated from the error signal. This duty ratio is then sent to the PWM. For the purpose of controlling the flow of electricity into or out of the batteries, the pulse width modulation (PWM) may be used to generate the switching pulses for the battery switches (S1 and S2). While this is going on, the HF component can be calculated as follows:

14 $$\:\mathrm{I}\mathrm{HF\_ref}\text{}\mathrm{=}\text{}\mathrm{I}\mathrm{tot\_ref}\text{}-\mathrm{I}\mathrm{B\_ref}$$

The battery’s slow reaction time may prevent it from promptly aligning with reference current (I_B_ref_). Consequently, the control method accommodates this delay by determining the uncompensated battery power, which is articulated as:

15 $$\:\mathrm{P}\mathrm{B\_UC\:}\mathrm{=}\mathrm{\:(}\mathrm{I}\mathrm{HF\_ref}\text{}\mathrm{+}\text{}\mathrm{I}\mathrm{B\_err}\mathrm{)\:}\mathrm{*}\mathrm{\:V}\mathrm{B}$$

The control approach uses Eq. (16) to set a reference current for the SC in order to equalize the uncompensated battery power.

16 $$\:\mathrm{I}\mathrm{SC\_ref}=\frac{\mathrm{P}\mathrm{B\_UC}}{\mathrm{V}\mathrm{s}\mathrm{C}}=\mathrm{(}\mathrm{I}\mathrm{HF\_ref}\mathrm{\:\:+}\text{}\mathrm{I}\mathrm{B\_err}\mathrm{)}\mathrm{*}\:\frac{\mathrm{V}\mathrm{B}}{\mathrm{V}\mathrm{SC}}$$

The fundamental step in the control procedures is achieved by comparing (I_SC_ref_) with the actual I_SC_. Any error resulting from the two previously stated signals is thereafter managed by the fuzzy controller and 2DOF-PI, which generates the relevant D_SC_ depending on the error signal, subsequently relayed to the PWM generator. The PWM generator is responsible for producing switching pulses that are in sync with the switches of the SCs (S3 and S4). This allows the PWM generator to effectively regulate the power delivered or consumed by SCs. Through the process of modifying the duty cycle in response to the error signal, the control technique has the potential to guarantee that the actual current of the SCs is in accordance with the reference current and that an equitable distribution of power is maintained over the load.

### The proposed optimization algorithm

The Hippopotamus Optimization Algorithm (HOA) is a population-based metaheuristic inspired by the social organization and defensive behaviors of hippopotamuses in their natural habitats. Hippos typically form structured groups consisting of a dominant male, females, and calves, and they exhibit distinct responses such as confrontation and rapid escape when threatened. These behavioral patterns are abstracted in HOA into three main phases that guide the exploration and exploitation processes. Accordingly, candidate solutions (hippopotamuses) are initialized and iteratively updated within the search space based on position update rules, as formulated in Eq. (17)^71^.

17 $$\:{\mathrm{X}}_{\mathrm{hi}}\mathrm{=L}{\mathrm{L}}_{\mathrm{j}}\mathrm{+r}\mathrm{⋅}\left(\mathrm{U}{\mathrm{L}}_{\mathrm{j}}-\mathrm{L}{\mathrm{L}}_{\mathrm{j}}\right)\text{,\hspace{1em}}\mathrm{i}\text{=1,2,…,N,\hspace{1em}j=1,2,…,M}$$

where L_Lj_ and U_Lj_ specify the bottom and upper bounds of the j^th^ decision variable, and X_hi_ indicates the location of the hi^th^ candidate solution. r is random number between 0 and 1, N represents the overall population size inside the herd, and M is the total number of decision factors.

#### Phase 1: the update on the positioning of hippopotamuses in the river or pond (Exploration)

Using the known CF, the dominating hippopotamus or herd leader is chosen at this stage, and the herd is protected from danger by the prevailing solution. Once they reach maturity, male hippos are kicked out of the herd by the dominant male. From that point on, they have to find a way to establish their own dominance, which is outlined in Eq. (18).

18 $$\:{\mathrm{X}}_{\mathrm{i}}^{\mathrm{Mhippo}}\mathrm{=}{\mathrm{x}}_{\mathrm{i,j}}\text{}\mathrm{+}\text{}{\mathrm{y}}_{\mathrm{1}}\mathrm{⋅}\left({\mathrm{D}}_{\mathrm{hippo}}-{\mathrm{I}}_{\mathrm{1}}{\mathrm{x}}_{\mathrm{i,j}}\right)$$

$$\:\mathrm{for\:}\mathrm{i}=\mathrm{1,2},\dots\:,\lceil \frac{\mathrm{N}}{\mathrm{2}}\rceil\mathrm{\:and\:}\mathrm{j}=\mathrm{1,2},\dots\:,\mathrm{M}$$

19 $$\:h\mathrm{=}\left\{\begin{array}{c}\begin{array}{c}\begin{array}{c}{\mathrm{I}}_{2}\times {\overrightarrow{\mathrm{r}}}_{\mathrm{1}}+{\mathrm{(Q}}_{1})\\\:\mathrm{2}\times{\overrightarrow{\mathrm{r}}}_{2}-\mathrm{1}\\\:{\overrightarrow{\mathrm{r}}}_{\mathrm{3}}\end{array}\\\:{\mathrm{I}}_{1}\times {\overrightarrow{\mathrm{r}}}_{4}+{\mathrm{(Q}}_{2})\end{array}\\\:{\overrightarrow{\mathrm{r}}}_{\mathrm{5}}\end{array}\right.$$

Here, D_hippo_ denotes the location of the dominant hippopotamus, X_i_^Mhippo^ denotes the position of the male hippopotamus, y_1_ is a random value between 0 and 1, and I_1_, I_2_ are integer integers between 1 and 2. Vectors r_1_, r_2_, r_3_, and r_4_ are randomly created within the range of 0 to 1, whereas r_5_ is a random number also between 0 and 1. Q_1_ and Q_2_ are random integers, either 0 or 1.

The behavior of female and juvenile hippopotamuses is influenced by two random vectors, h_1_ or h_2_, derived from five distinct circumstances as stated in the Eq. (19)^71^.

#### Phase 2: hippopotamus defense mechanisms against predators (Exploration)

Hippopotamuses inhabit herds for protection, using their bulk to dissuade predators; nevertheless, juvenile and ailing members remain susceptible. Their principal defense mechanism involves facing the predator and emitting loud vocalizations to repel dangers. Equation (20) delineates the protective distance between the predator and the hippopotamus, whereas Eq. (21) illustrates the processes of evasion and predation.

20 $$\:\overrightarrow{\mathrm{D}}\mathrm{=}\left|{\mathrm{Predator}}_{\mathrm{j}}\mathrm{-}{\mathrm{x}}_{\mathrm{i,j}}\right|$$

21 $${\mathrm{X}}_{\mathrm{i}}^{\mathrm{Rhippo}}=\left\{\begin{array}{c}\overrightarrow{\mathrm{RL}}+{\mathrm{Predator}}_{\mathrm{j}}+\left(\frac{\theta}{\mathrm{c-d}\times \mathrm{cos}\left({2 \pi g}\right)}\right)\cdot \left(\frac{\mathrm{1}}{\mathrm{D}}\right)\:\:\:\:\:\:\:\:\:\:\:\:\:\:\:\:\:\:\:\:\:\:\:\:\:\:{\mathrm{F}}_{{\mathrm{Predator}}_{\mathrm{j}}}<{\mathrm{F}}_{\mathrm{i}}\\\:\overrightarrow{\mathrm{RL}}+{\mathrm{Predator}}_{\mathrm{j}}+\left(\frac{\theta}{\mathrm{c-d}\times \mathrm{cos}\left({2\pi g}\right)}\right)\cdot\left(\frac{1}{2 \times \overrightarrow{\mathrm{D}}+\overrightarrow{{\mathrm{r}}_{\mathrm{9}}}}\right)\:\:\:\:\:\:\:\:\:\:\:\:\:\:\:\:\:{\mathrm{F}}_{{\mathrm{Predator}}_{\mathrm{j}}}\geq{\mathrm{F}}_{\mathrm{i}}\end{array}\right.$$

where X_i_^Rhippo^ indicates the hippopotamus’s position relative to the predator, $\:\overrightarrow{RL}$ signifies a random vector following a Lévy distribution, ϑ is a random variable that varies between 2 and 4, while c and d are random variables limited to the intervals [1, 1.5] and^2,3^, respectively. g is a uniformly distributed random value within the interval of -1 to 1, whereas $\:\overrightarrow{{r}_{9}}$ denotes a random vector.

#### Phase 3: hippopotamus evading the predator (Exploitation)

Since predators like lions and hyenas tend to stay away from water, a hippopotamus will typically seek refuge near a body of water if it is attacked by multiple enemies or is unable to fight them off. This method improves local search utilization in the HOA model, as delineated in Eqs. (22) and (23).

22 $$\:\mathrm{L}{\mathrm{L}}_{\mathrm{j}}^{\mathrm{local}}\mathrm{=}\frac{\mathrm{L}{\mathrm{L}}_{\mathrm{j}}}{\mathrm{iter}}\mathrm{,}{\hspace{1em}}\mathrm{U}{\mathrm{L}}_{\mathrm{j}}^{\mathrm{local}}\mathrm{=}\frac{\mathrm{U}{\mathrm{L}}_{\mathrm{j}}}{\mathrm{iter}}\mathrm{,}{\hspace{1em}}\mathrm{iter}\mathrm{=1,2,…,}{\mathrm{iter}}_{\mathrm{max}}$$

23 $$\:{\mathrm{X}}_{\mathrm{i}}^{{\mathrm{H}}_{\mathrm{Hippo}}}\mathrm{=}{\mathrm{X}}_{\mathrm{ij}}\mathrm{+}{\mathrm{r}}_{\mathrm{10}}\mathrm{⋅}\left(\mathrm{L}{\mathrm{L}}_{\mathrm{j}}^{\mathrm{local}}\mathrm{+{\alpha}}\left(\mathrm{U}{\mathrm{L}}_{\mathrm{j}}^{\mathrm{local}}-\mathrm{L}{\mathrm{L}}_{\mathrm{j}}^{\mathrm{local}}\right)\right)$$

**Fig. 7:**
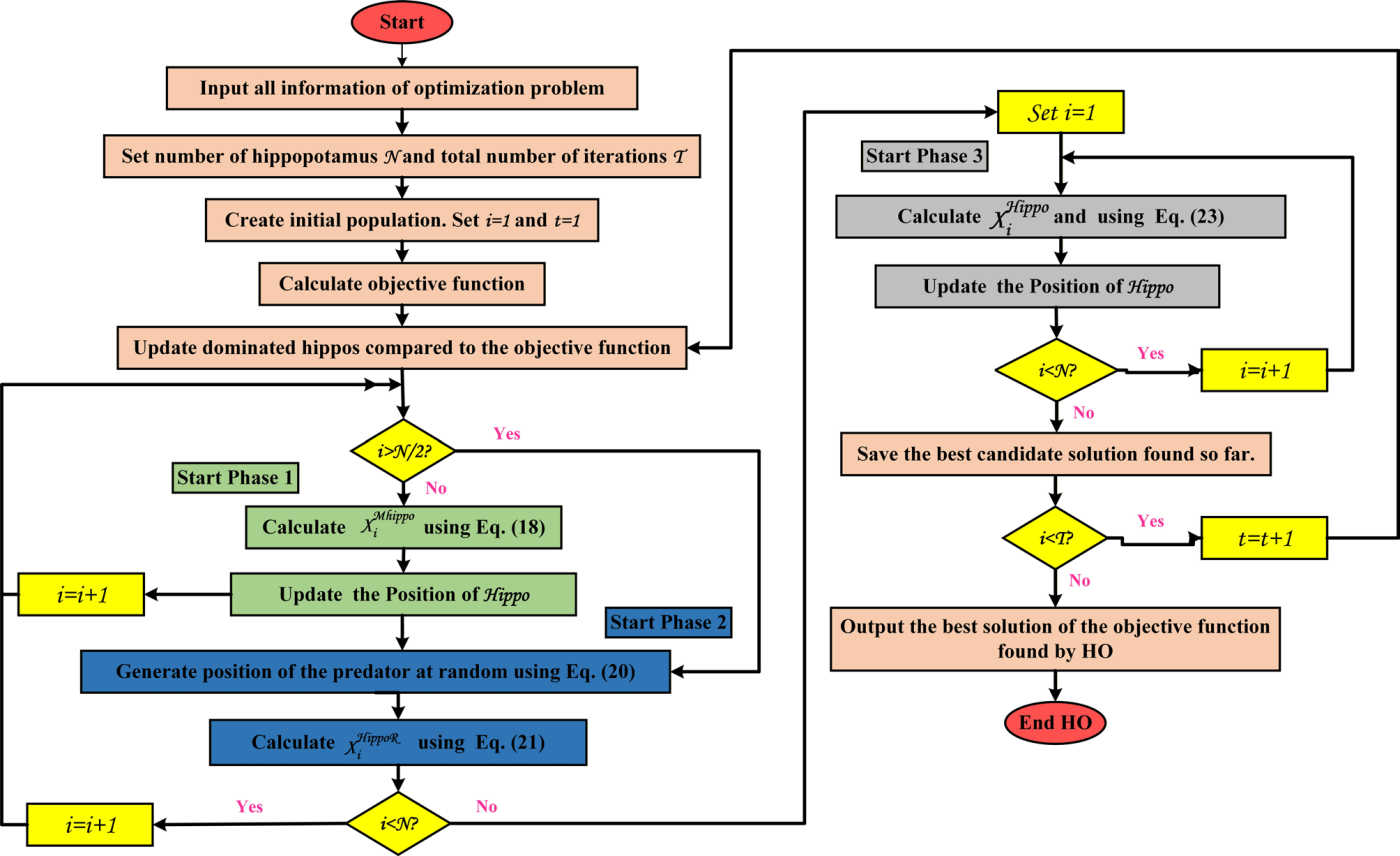
Flowchart of the HO optimizer.

$\:{X}_{i}^{{H}_{Hippo}}$ denotes the location of the hippopotamus in pursuit of the nearest secure area, constrained by the lower and upper limits: $\:\mathrm{L}{\mathrm{L}}_{\mathrm{j}}^{\mathrm{local}}$and $\:\mathrm{U}{\mathrm{L}}_{\mathrm{j}}^{\mathrm{local}}$, respectively. iter represents the current iteration, while $\:{\mathrm{iter}}_{\mathrm{max}}$ signifies the total number of HOA iterations; $\:\mathrm{{\alpha}}$ and r_10_ are randomly generated vectors. The HOA process flow is shown in Fig. 7.

### Control system design

#### Fuzzy logic controller

**Fig. 8:**
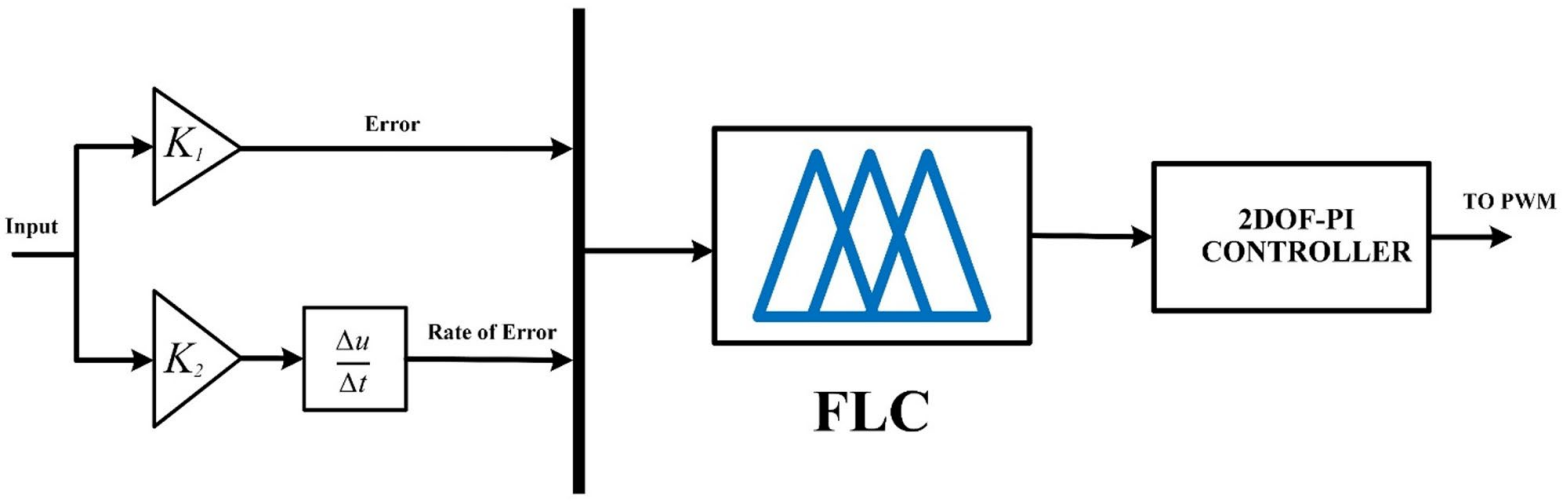
Configuration of Fuzzy Logic with 2DOF-PI Controller.

Fuzzy logic was chosen as the control architecture for managing both DC/DC converters due to its capability to operate effectively without requiring an exact mathematical model or transfer function of the system, thereby simplifying the design process and enhancing adaptability. Its inherent tolerance to imprecise or uncertain input data makes it highly robust under varying operating conditions and system nonlinearities. Furthermore, FLCs have been shown to deliver performance levels comparable to those of conventional PI or PID controllers, while offering improved flexibility in handling complex, nonlinear, and time-varying systems. This makes fuzzy logic a suitable and reliable control strategy for achieving stable and efficient power management in DC/DC converter applications^72^. The FLC structure with 2DOF-PI is illustrated in Fig. 8.

**Fig. 9:**
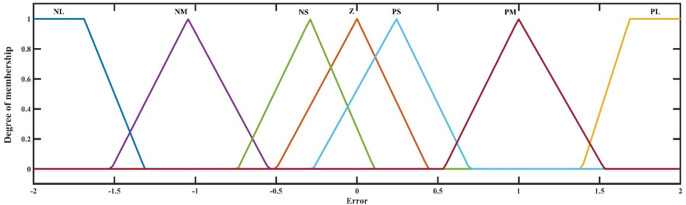
Error membership function.

For the two different inputs to the controller, two input membership functions are required. Membership functions are clear curves that define the correspondence between each input value and a certain value, or the degree of truth related to that value. The preliminary membership function is the error as seen in Fig. 9.

**Fig. 10:**
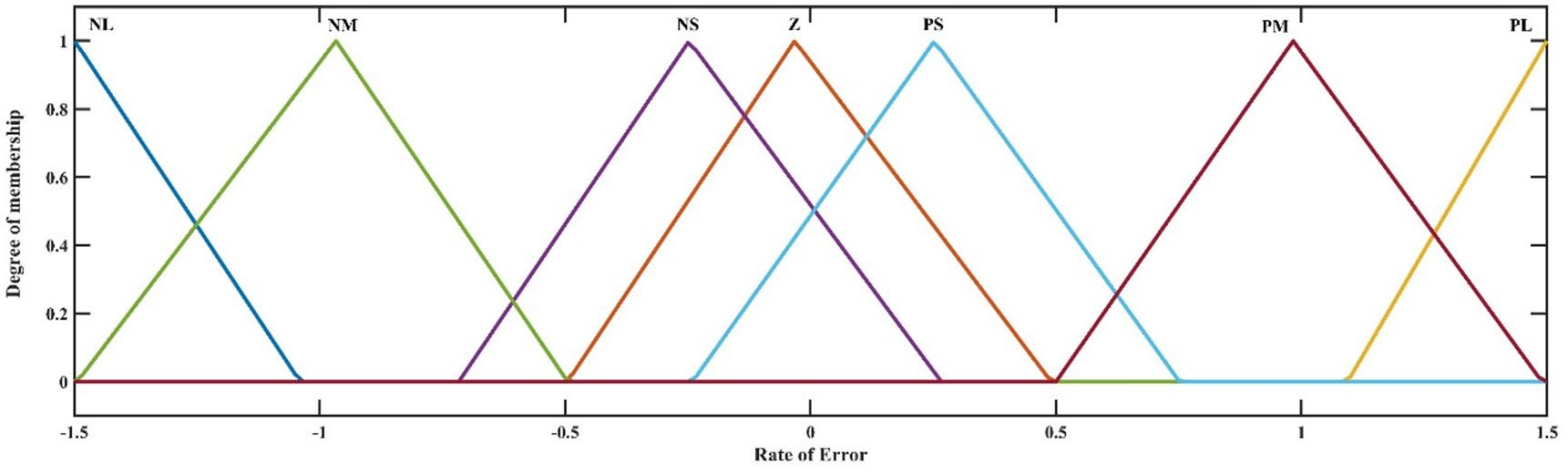
Rate of Error membership function.

The error membership function’s rate of change is represented by the second membership function, as shown in Fig. 10. This function assesses whether the mistake diminishes at an acceptable rate.

**Fig. 11:**
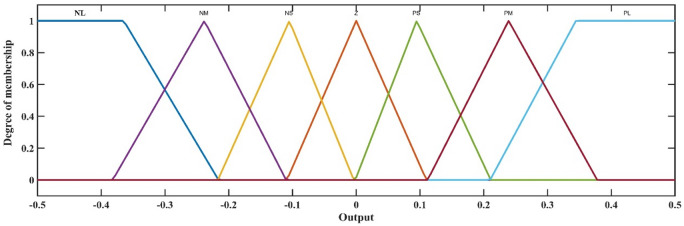
Output membership function.

Zero (Z), positive small (PS), positive medium (PM), negative large (PL), negative medium (NM), negative small (NS), and negative large (NL) are the seven categories that make up each membership function. Due to its singular output, the FLC requires just one output membership function. Figure 11 shows the membership function that was produced.

**Fig. 12:**
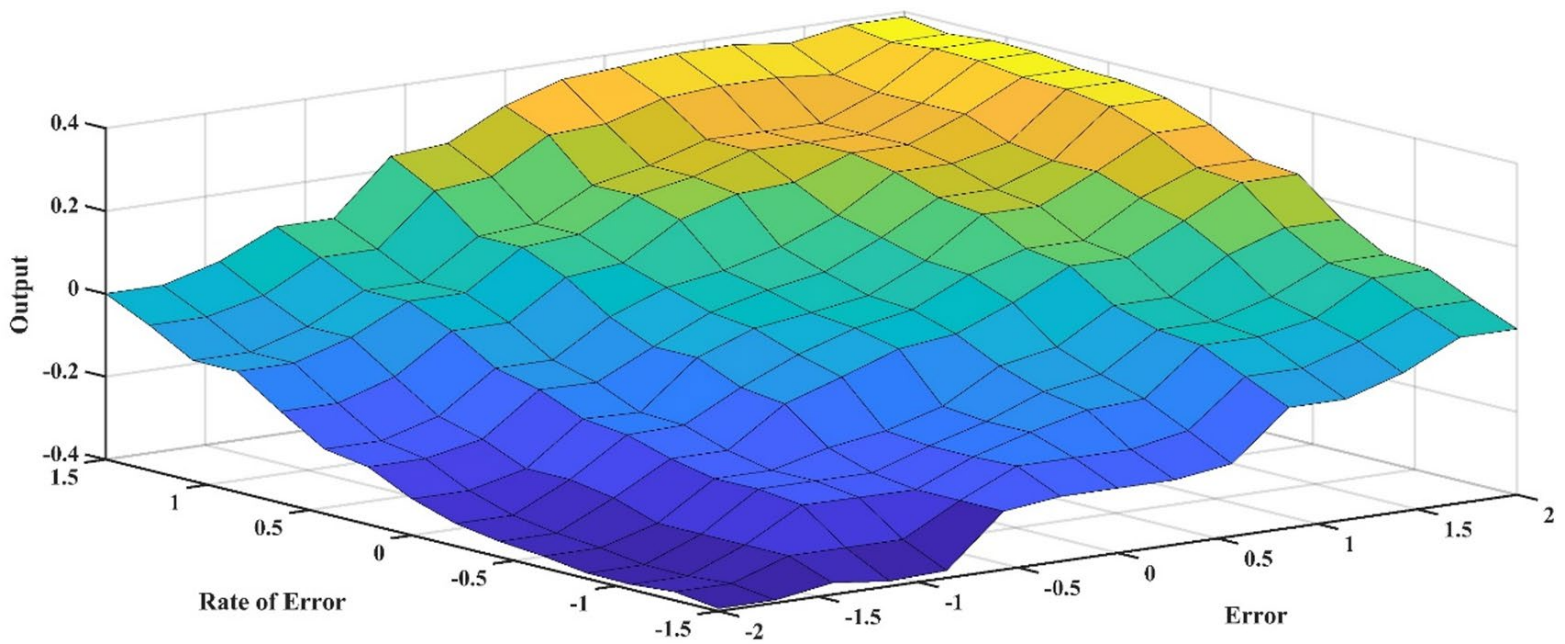
FLC Rule Surface Viewer.

Throughout the simulation process, the membership functions’ input ranges were modified until the controller functioned as intended. Gain and, conversely, input function sensitivity can be changed by adjusting the membership functions’ input range. The suggested fuzzy logic rules are delineated in Table 4 below. The regulations were instituted to guarantee that the controller evaluates both the deviation between the measured value and the reference value and, by examining the error’s derivative, determines if the error is decreasing at an appropriate rate, thereby adjusting the duty cycle as necessary. FLC utilized the maximum method for aggregation and the centroid technique for defuzzification. The Mamdani inference method was employed^49^. Figure 12 illustrates the control surface that delineates the input-output correlation of the (FLC). Determining the appropriate input and output values and configurations for FLC is a formidable problem.

**Table 4:** Fuzzy logic rule table.

∆Error								
Error		NL	NM	NS	Z	PS	PM	PL
	**NL**	NL	NL	NL	NM	NM	NS	Z
	**NM**	NL	NM	NM	NS	NS	Z	PS
	**NS**	NM	NM	NS	NS	Z	PS	PM
	**Z**	NM	NS	NS	Z	PS	PS	PM
	**PS**	NM	NS	Z	PS	PS	PM	PL
	**PM**	NS	Z	PS	PS	PM	PM	PL
	**PL**	Z	PS	PM	PM	PL	PL	PL

#### 2DOF-PI controller

**Fig. 13:**
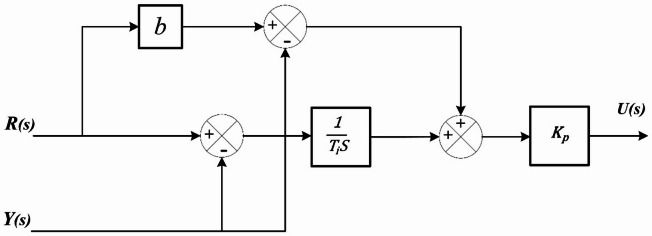
Structure of 2DOF-PI controller.

The proposed controller integrates the advantages of Fuzzy logic with 2DOF-PI controllers, resulting in enhanced power regulation. The 2DOF-PI controller configuration mirrors that of the PI controller, including an additional weight component to the reference elements. Figure 13 illustrates the configuration of the 2DOF-PI regulator. Equation (24) is the transfer function of the 2DOF-PI controller^73^.

24 $$\:\mathrm{U(s)=}{\mathrm{K}}_{\mathrm{p}}\text{}\mathrm{[(}\mathrm{b}\text{}\mathrm{R(s)}\text{}-\text{}\mathrm{Y(s)}\text{}\mathrm{)}\text{}\mathrm{+}\frac{\mathrm{1}}{{\mathrm{T}}_{\mathrm{i}}\mathrm{S}}\text{}\mathrm{(R(s)}\text{}-\mathrm{Y(s))}\text{}\mathrm{]}$$

b represents the proportionate set-point weighting adjustment.

The system parameters are constrained as follows:

$$\:\left\{\begin{array}{c}{KP}_{min}\le\:KP\le\:{KP}_{max}\\\:{KI}_{min}\le\:KI\le\:{KI}_{max}\\\:{b}_{min}\le\:b\le\:{b}_{max}\\\:{k1}_{min}\le\:k1\le\:{k2}_{max}\\\:{k2}_{min}\le\:k2\le\:{k2}_{max}\end{array}\right.$$

A suggested controller is intended to distribute power between the battery and the SC. The cost function $\:J\:$is now defined as the Integral of Squared Error (ISE) of the main HESS control variables and is given by:

25 $$\:J={\int\:}_{0}^{Tsim}\left.\left[{\stackrel{-}{e}}_{{V}_{dc}}^{2\left(t\right)}+{\stackrel{-}{e}}_{{I}_{SC}}^{2\left(t\right)}+{\stackrel{-}{e}}_{{I}_{B}}^{2\left(t\right)}\right.\right]dt$$

where $\:{\stackrel{\prime }{e}}_{Vdc}$, $\:{\stackrel{\prime }{e}}_{ISC}$, and $\:{\stackrel{\prime }{e}}_{IB}$denote the normalized DC-bus voltage error, supercapacitor current error, and battery current error, respectively. These variables represent the key performance indicators governing DC-link stability, transient power compensation, and battery current regulation within the hybrid energy storage system.

## Simulation results and discussion

**Fig. 14:**
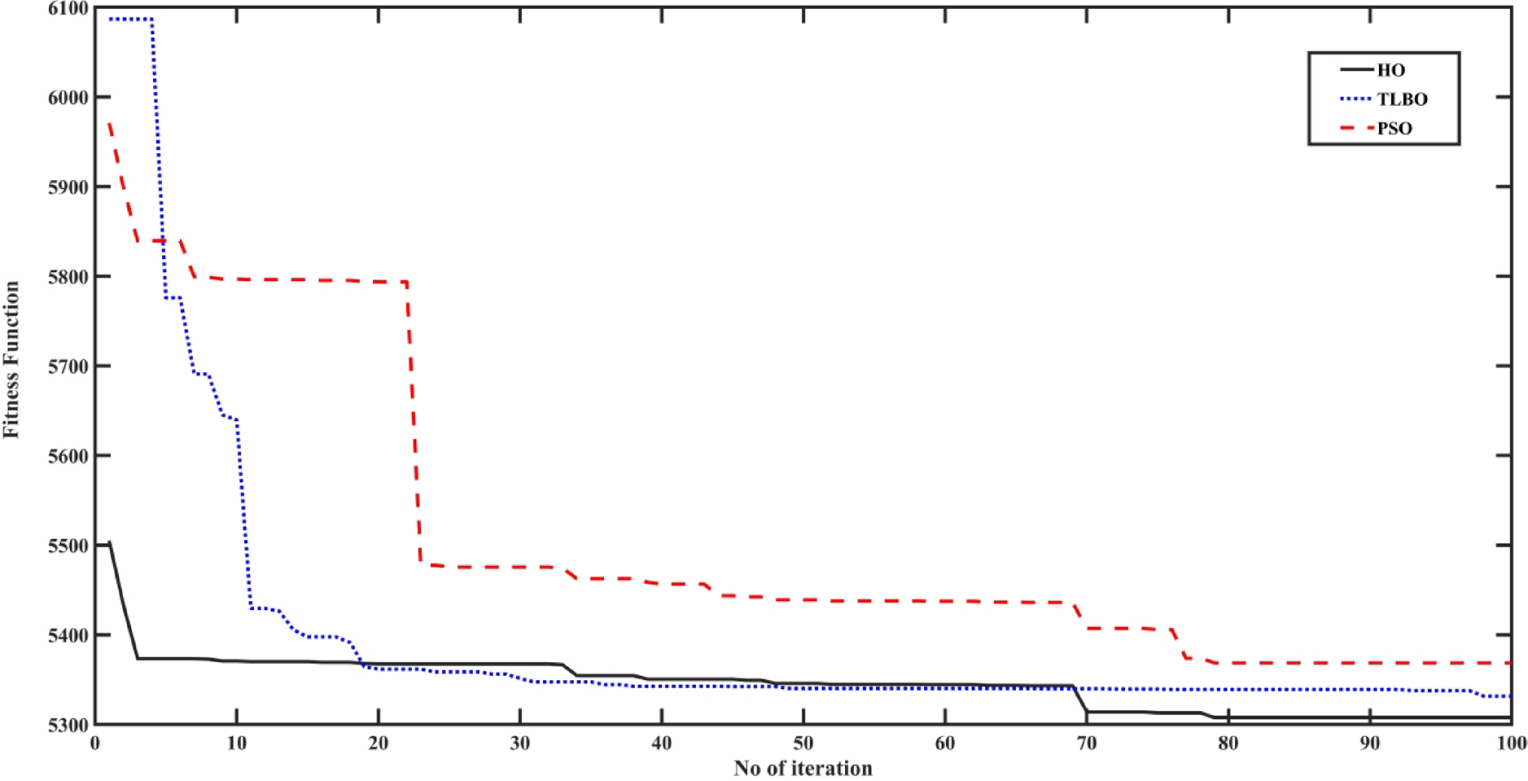
Convergence rate of the three optimization techniques.

This section verifies the constructed Fuzzy 2DOF-PI based HO controller under varied load situations and fluctuations in solar irradiation. The simulations in this study are performed under idealized conditions, without explicitly modeling practical non-idealities such as converter switching losses, measurement noise, communication delays, SOC estimation errors or component aging. The main objective is a fair comparative evaluation of control strategies under identical assumptions to isolate the effect of the proposed method. The objective is to diminish peak power and extend battery life to comply with the state of charge limitations of the battery and SC by optimizing the controller settings. To assess the efficacy of Fuzzy with 2DOF-PI, a comprehensive comparison will be conducted between the fuzzy PI-based TLBO, PSO, and non-optimization fuzzy methods, including conventional PI. To examine its performance, the planned system has been simulated using the MATLAB/Simulink^®^ (2024b) environment. The convergence characteristics of the optimization algorithms HO, TLBO, and PSO are illustrated in Fig. 14. At the final iteration, the HO-based optimization achieves the lowest fitness value of 5307.7, compared to 5368.8 for TLBO and 5531.7 for PSO. This demonstrates that the HO-based offline parameter tuning not only converges more rapidly but also attains a higher-quality optimal solution, indicating superior exploitation capability and greater efficiency in tuning the proposed Fuzzy 2DOF-PI controller compared with the benchmark optimization algorithms. All algorithms are conducted based on 30 search populations and 100 iterations. Table 5 below lists the optimal values of the utilized controllers.

**Table 5:** The optimum parameters of the controllers in Case1.

Controller	DC BUS					SC Battery									
	KP_DC	KI_DC	bDC	K1_DC	K2_DC	KP_SC	KI_SC	bSC	K1_SC	K2_SC	KP_B	KI_B	bB	K1_B	K2_B
Conventional PI	1.62	674	–	–	–	0.875	1274.08	–	–	–	0.22	800	–	–	–
FPI- based PSO	2.09	1979	–	1.992	2.481	4.211	621.03	–	1.426	2.351	4.74	3595	–	1.972	0.392
FPI-based TLBO	1.91	1542	–	0.83	3.362	2.384	1247.25	–	1.333	1.428	3.732	2995	–	1.823	1.533
F-2DOFPI-based HO (Proposed)	3.18	1915	1.62	3.215	3.364	1.855	1284. 6	0.66	3.763	3.918	1.471	947.29	0.84	2.537	1.924

### Scenario 1: variation of solar irradiance

**Fig. 15:**
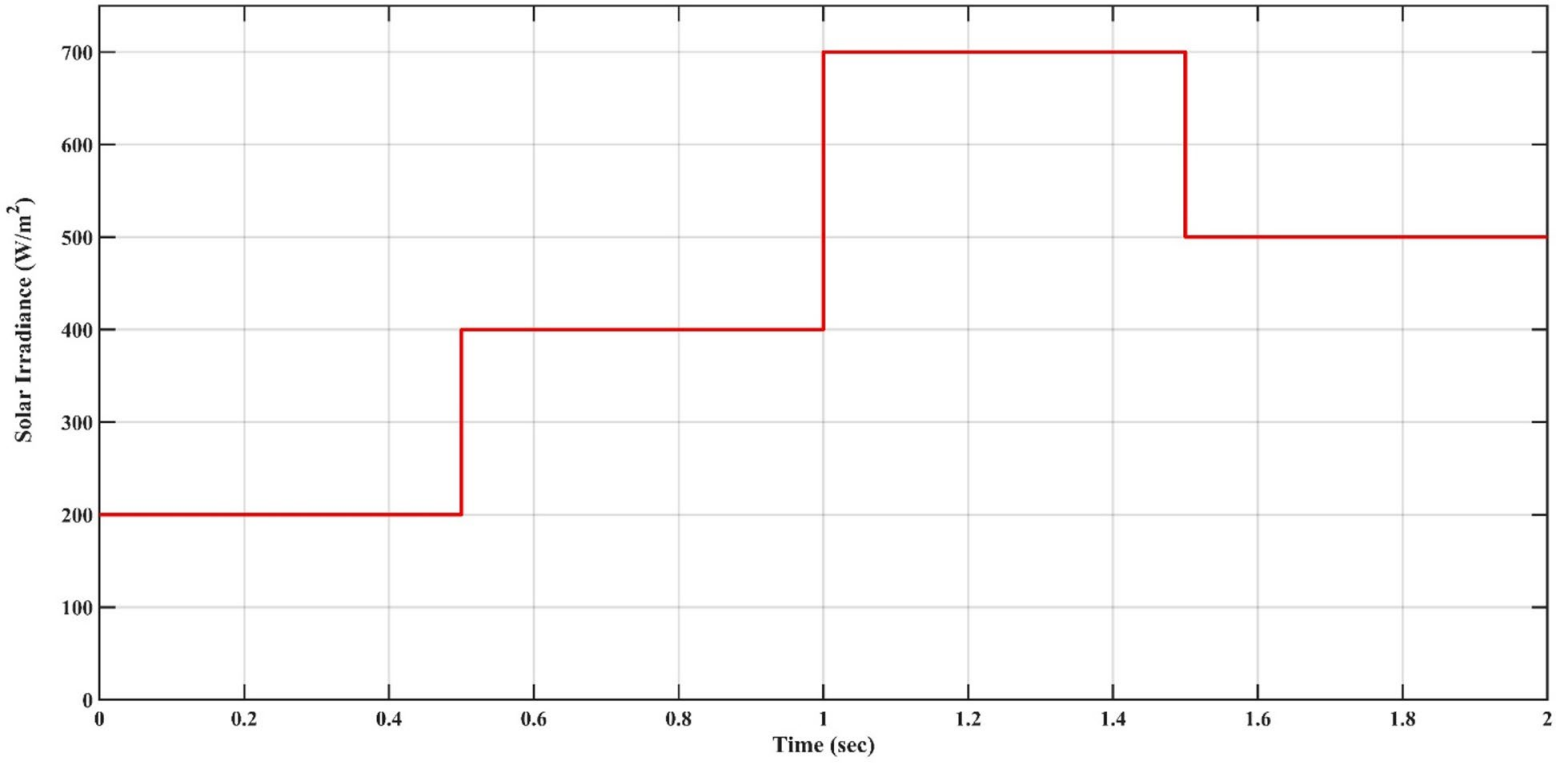
Solar Irradiance Variation.

**Fig. 16:**
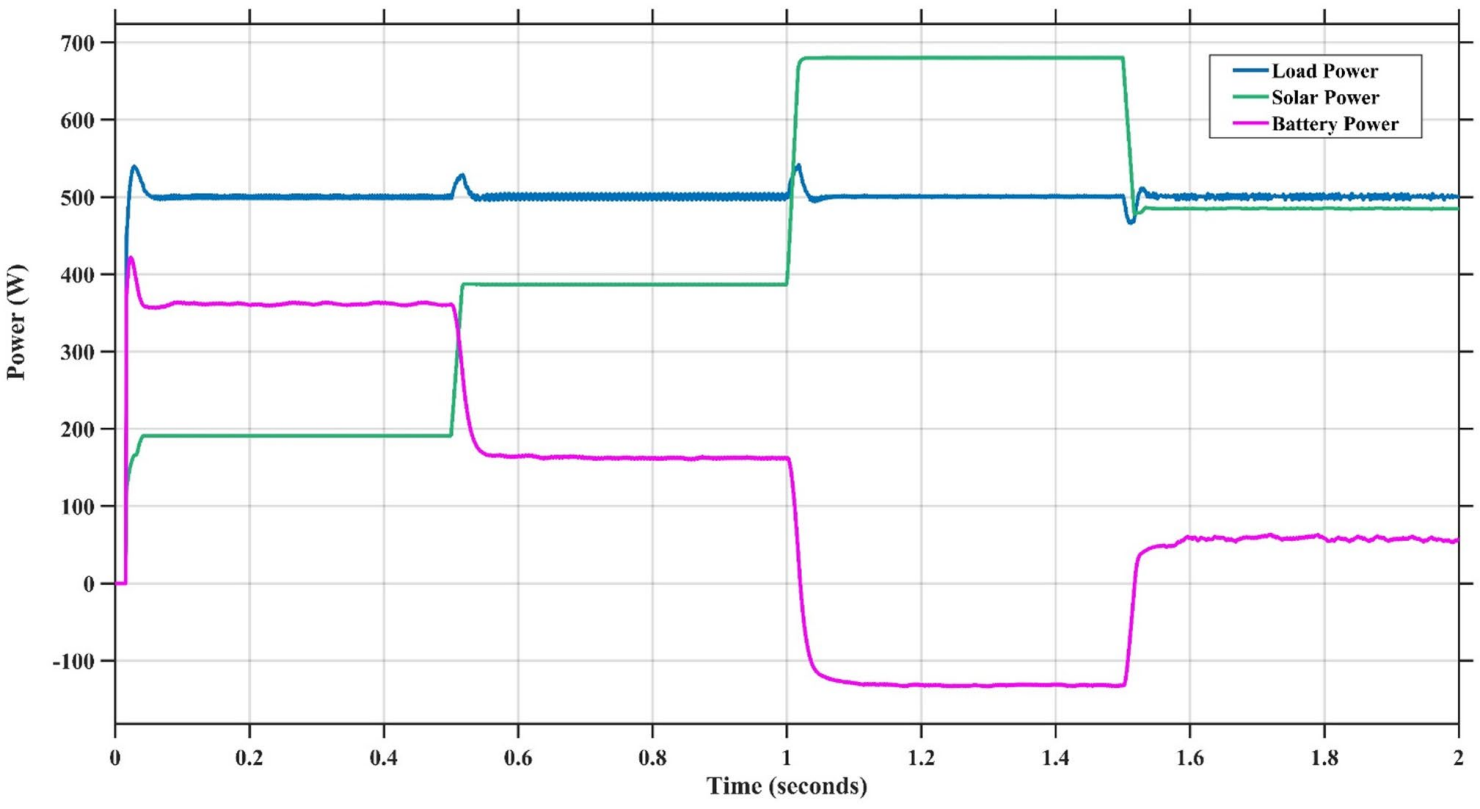
Power Responses of the Proposed Control Strategy.

**Fig. 17:**
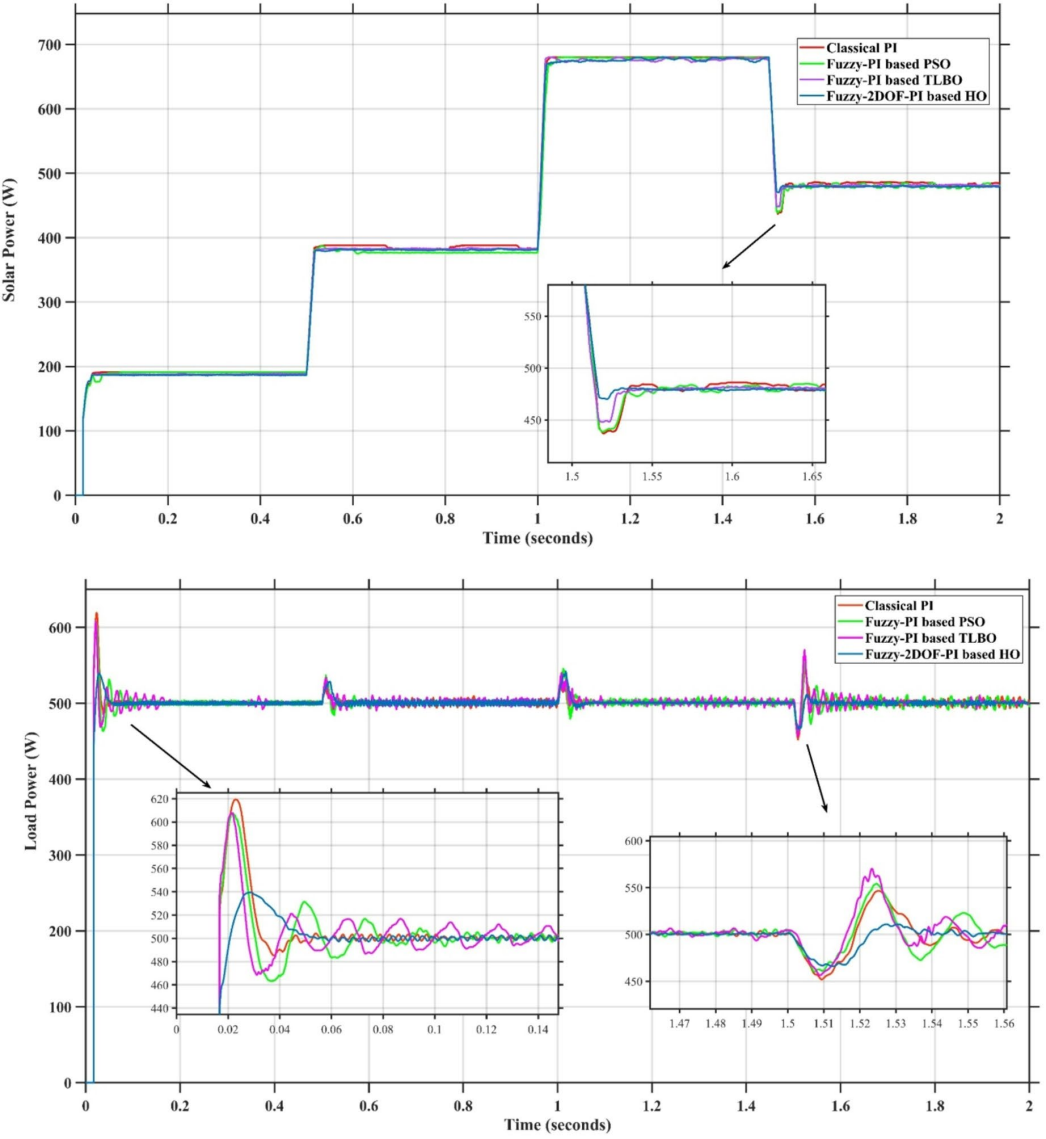
Responses of Solar and Load Powers for different controllers.

**Fig. 18:**
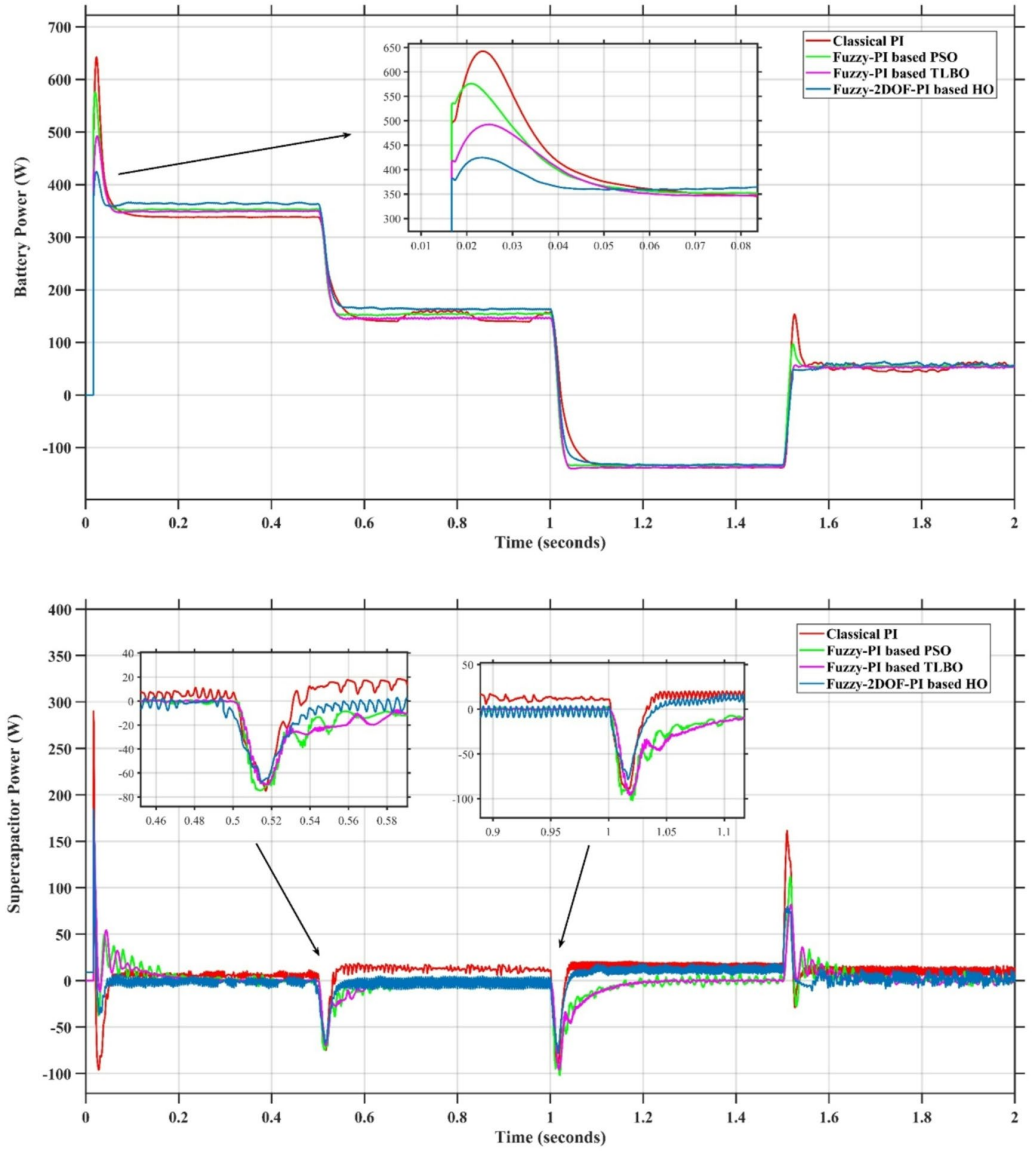
Responses of Battery and SC Powers for different controllers.

**Fig. 19:**
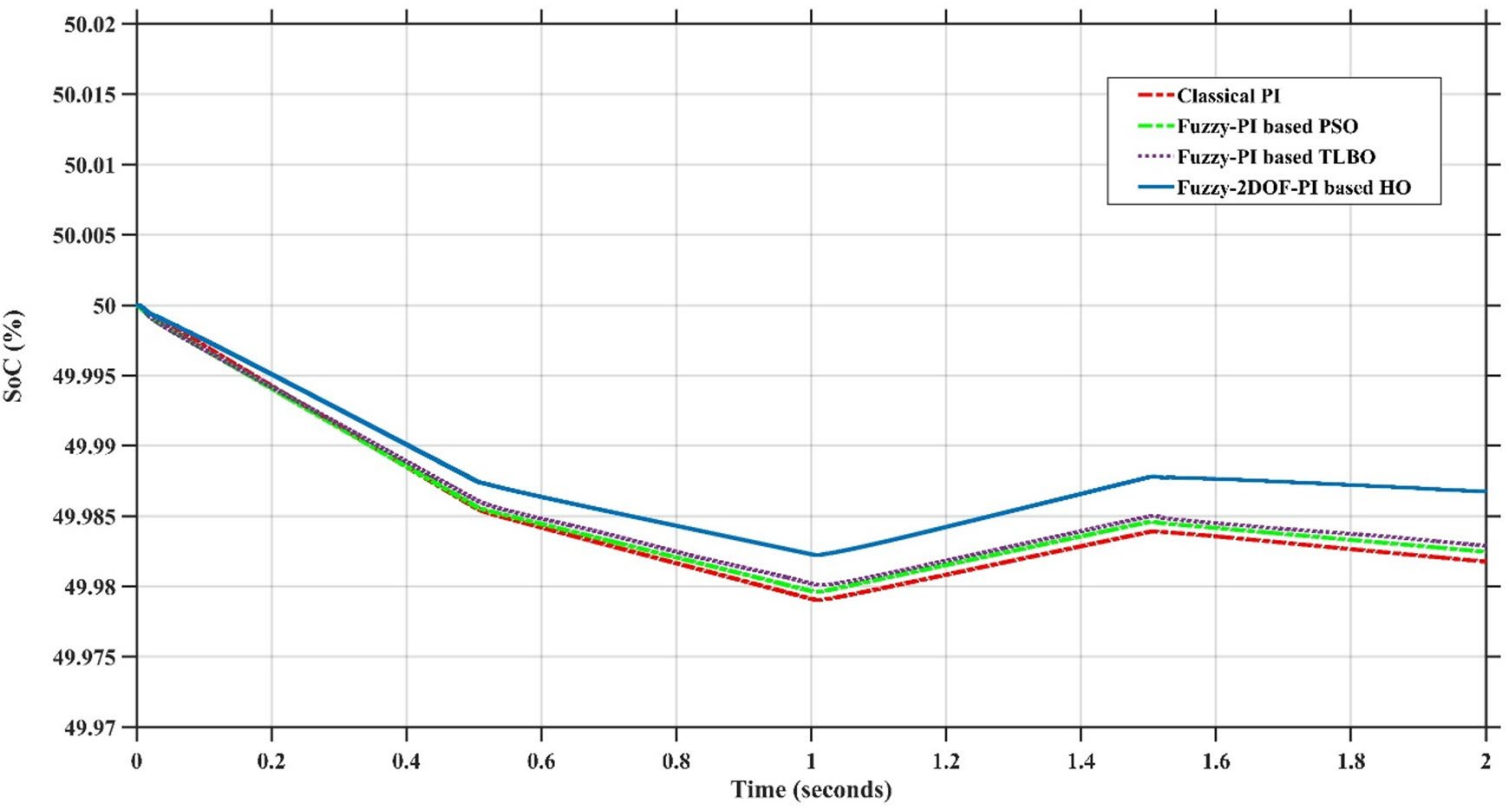
Battery State of Charge.

**Fig. 20:**
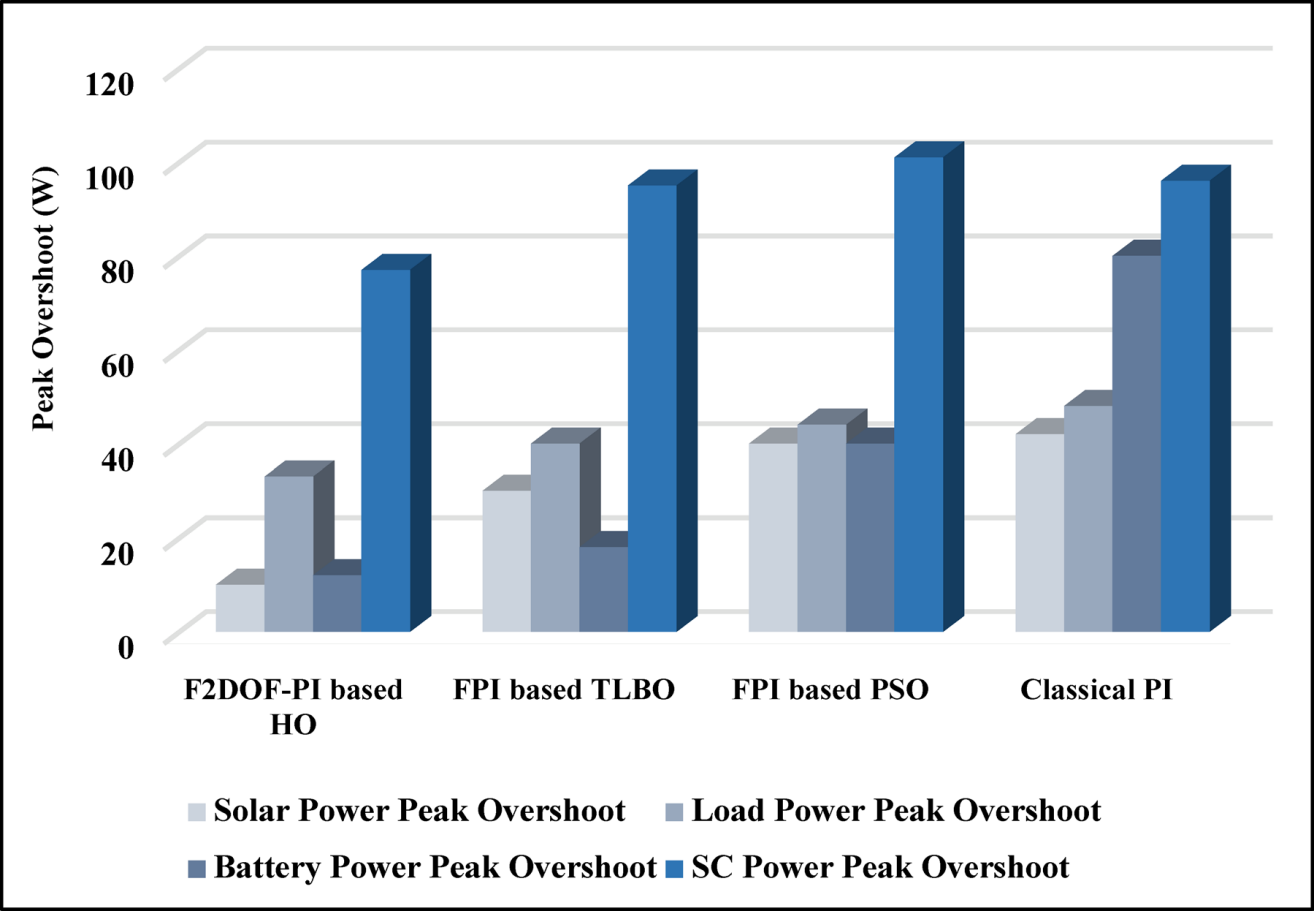
Peak overshoot for different controllers.

**Fig. 21:**
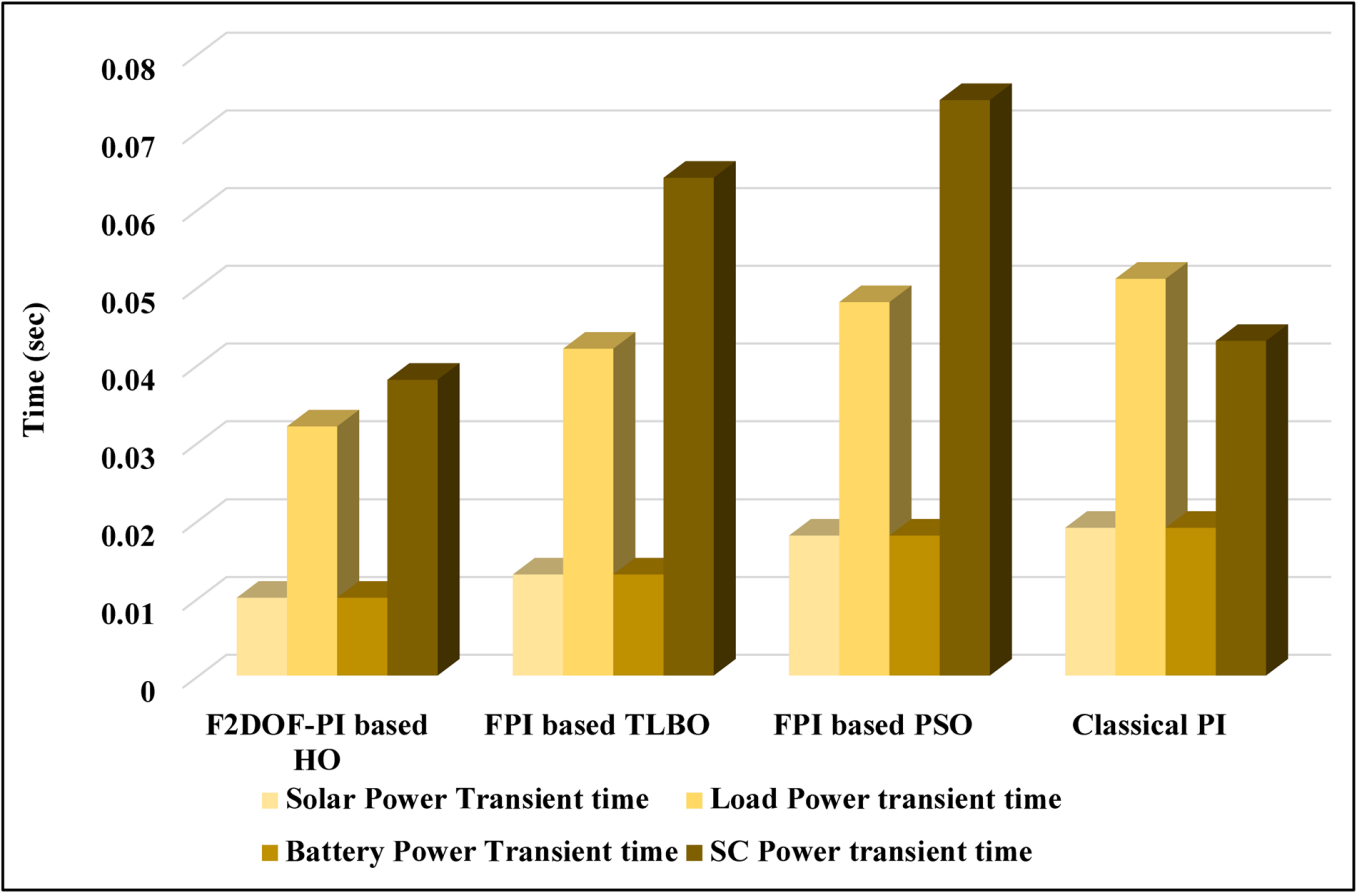
Transient time for different controllers.

In this case, the battery’s state of charge was originally at 50%. The PV system and HESS carry over the entire load requirement. Figure 15 illustrates how the amount of solar radiation is thought to fluctuate. The irradiance remains at 200 W/m² from 0 to 0.5 s, then increases to 400 W/m² from 0.5 to 1.0 s. At 1.0 s, there is a further increase to 700 W/m², maintained until 1.5 s. Subsequently, it decreases to 500 W/m² and remains stable for 2.0 s. This stepped irradiance profile is frequently employed to evaluate the dynamic response of photovoltaic systems and (MPPT) algorithms under fluctuating solar conditions, such as changing cloud cover or varied weather. The sudden alterations facilitate the assessment of tracking efficacy, control responsiveness, and system stability. The graph highlights how the battery and solar system work together to maintain a constant load power requirement by showing the power distribution fluctuations over time among the solar source, battery, and load. While the solar power production shows a stepwise increase in response to variations in sun irradiation, the load power stays roughly constant at 500 W over the 2-second interval. Initially, when there is not enough solar input, the battery makes up the difference by giving the load the extra power it needs. The battery contribution correspondingly decreases as solar power increases at approximately 0.5 and 1.0 s, demonstrating effective load distribution. Negative battery power levels, which indicate charging activity, occur when solar generation exceeds load demand during the peak solar irradiance period (roughly 1.0 to 1.5 s). When the amount of solar input decreases after 1.5 s, the battery switches back to discharging mode to make up for the lost solar generation and keep the load powered continuously. Figure 16 highlights the cooperative behavior of the battery and solar system in maintaining a constant load power demand by showing the dynamic power sharing between the solar source, battery, and load over time. Figures 17 and 18 depict the comparative analysis of power responses for various control strategies, including classical PI^62^, fuzzy PI based on TLBO^53^, fuzzy PI based on PSO^52^, and the proposed fuzzy 2DOF-PI based on HO. Figure 19 illustrates the battery state SoC. The peak overshoot and transient time for the various controllers are illustrated in Figs. 20 and 21, respectively. The comparative results of peak overshoot and transient time for the four control strategies clearly demonstrate that the F2DOF-PI based HO outperforms the other methods in both stability and dynamic response. The F2DOF-PI based HO achieves the lowest values across all power sources, with the battery power peak overshoot reduced by about 20% and the supercapacitor power peak overshoot lowered by nearly 23% compared to the classical PI controller. Meanwhile, the FPI-based TLBO and FPI-based PSO show moderate improvements over the classical PI, yet their overshoot levels remain considerably higher than those of the F2DOF-PI based HO. The proposed method also excels, reducing solar power transient time by approximately 40% and load power transient time by around 50% relative to the classical PI, which means it responds faster to system disturbances. Although the FPI-based TLBO and PSO exhibit some gains in transient performance compared to the classical PI, they still lag behind the F2DOF-PI based HO.

### Scenario 2: penetration of step load increase

**Fig. 22:**
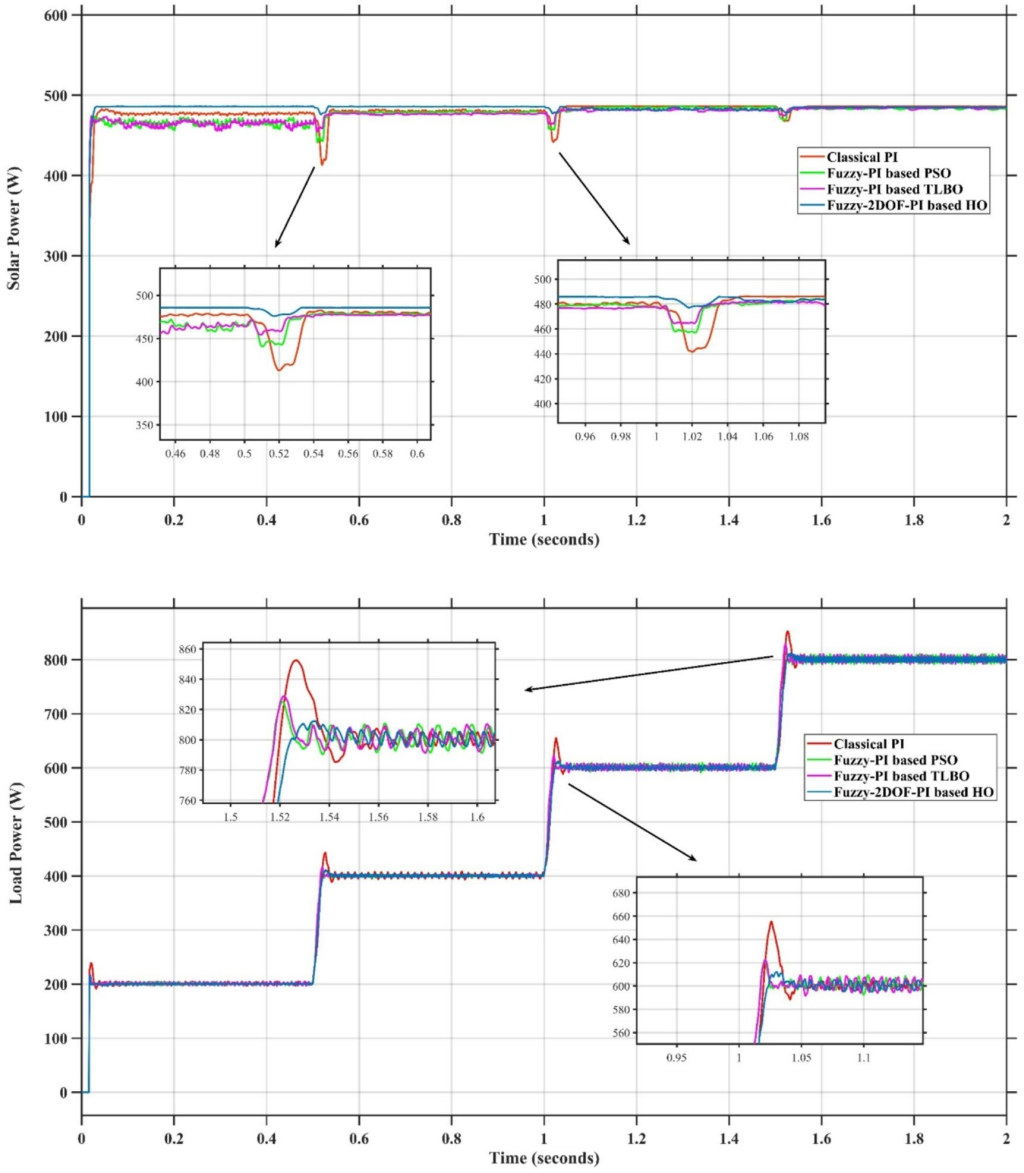
Responses of Solar and Load powers for different controllers.

**Fig. 23:**
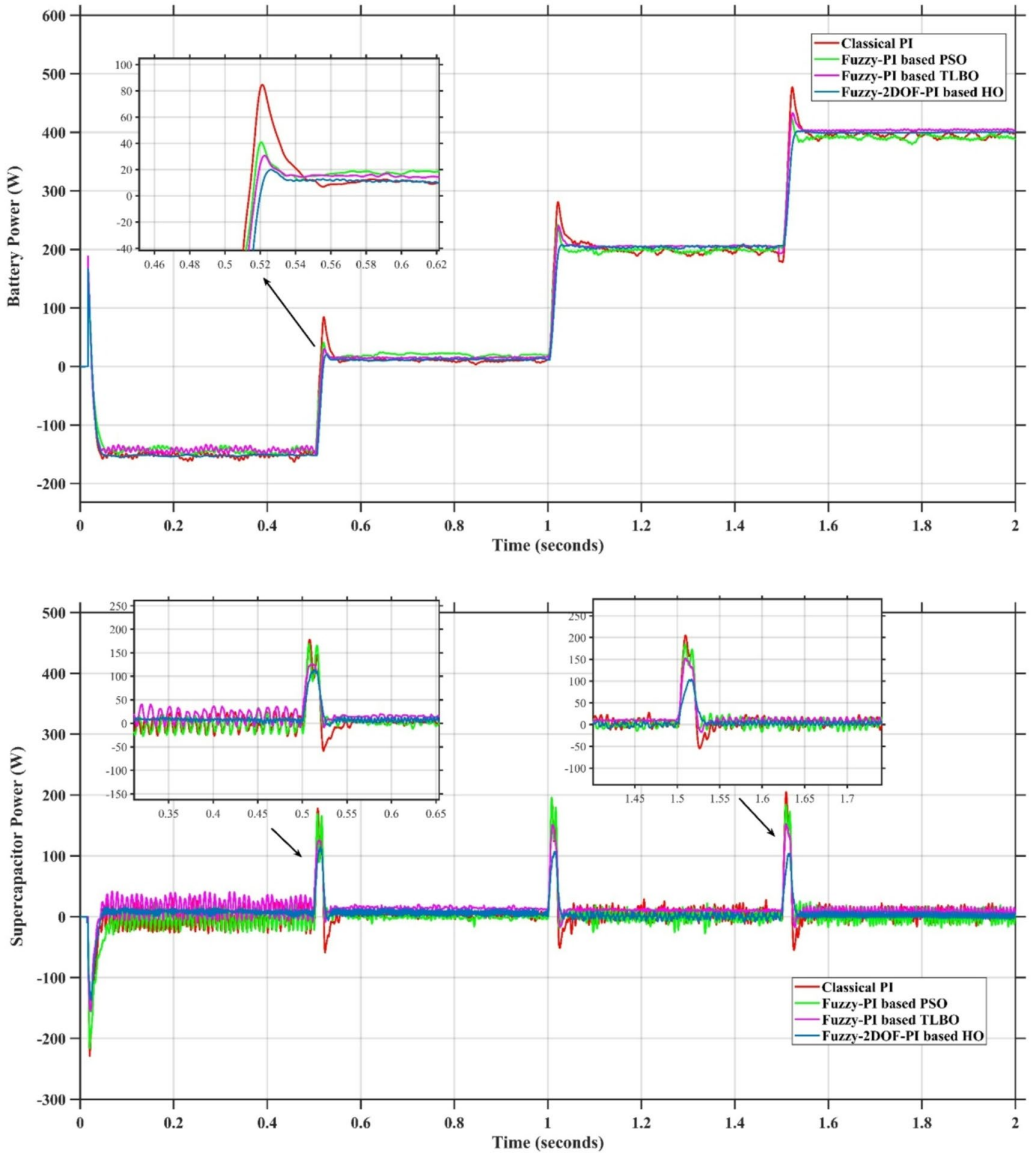
Responses of Battery and Supercapacitor Responses for different controllers.

**Fig. 24:**
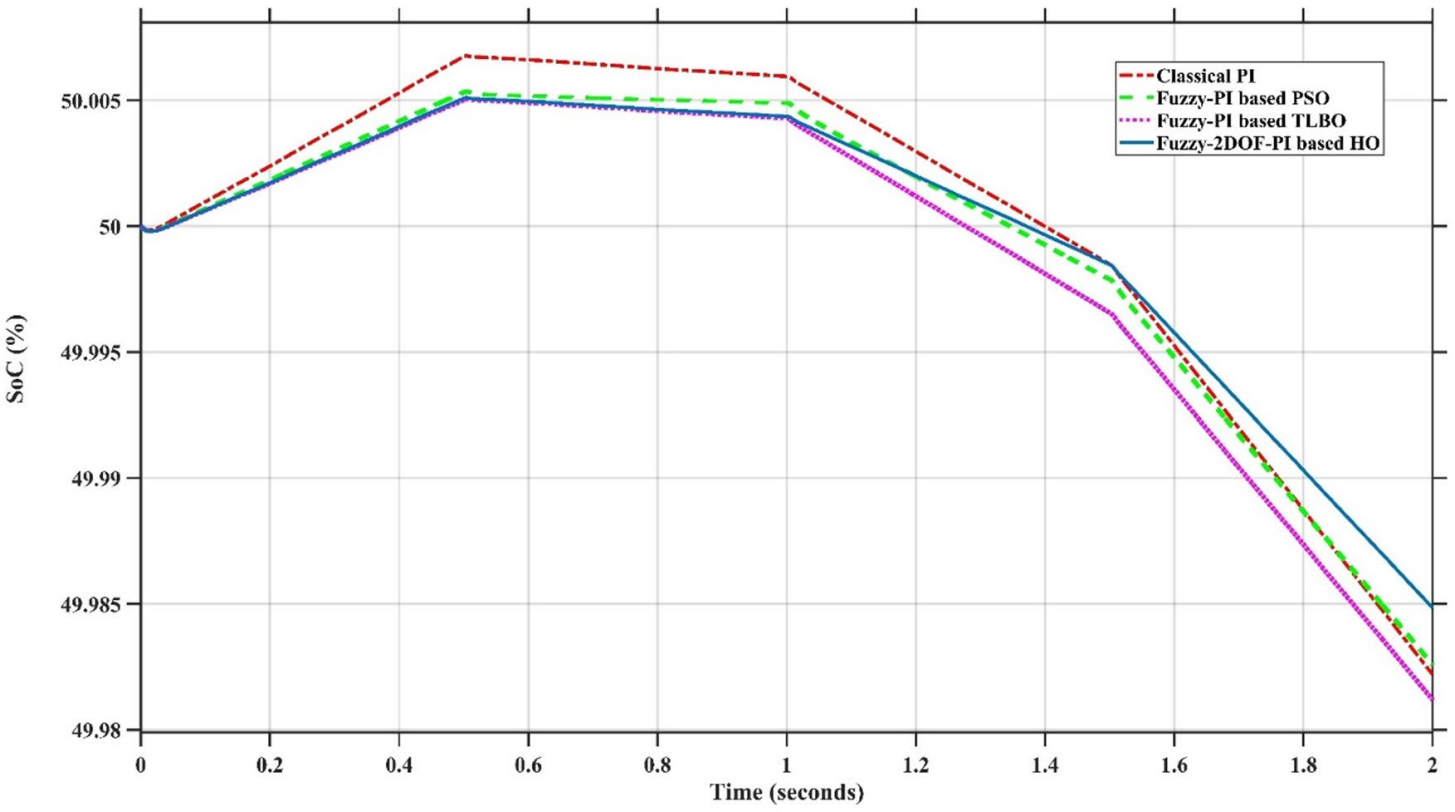
Battery State of Charge.

**Fig. 25:**
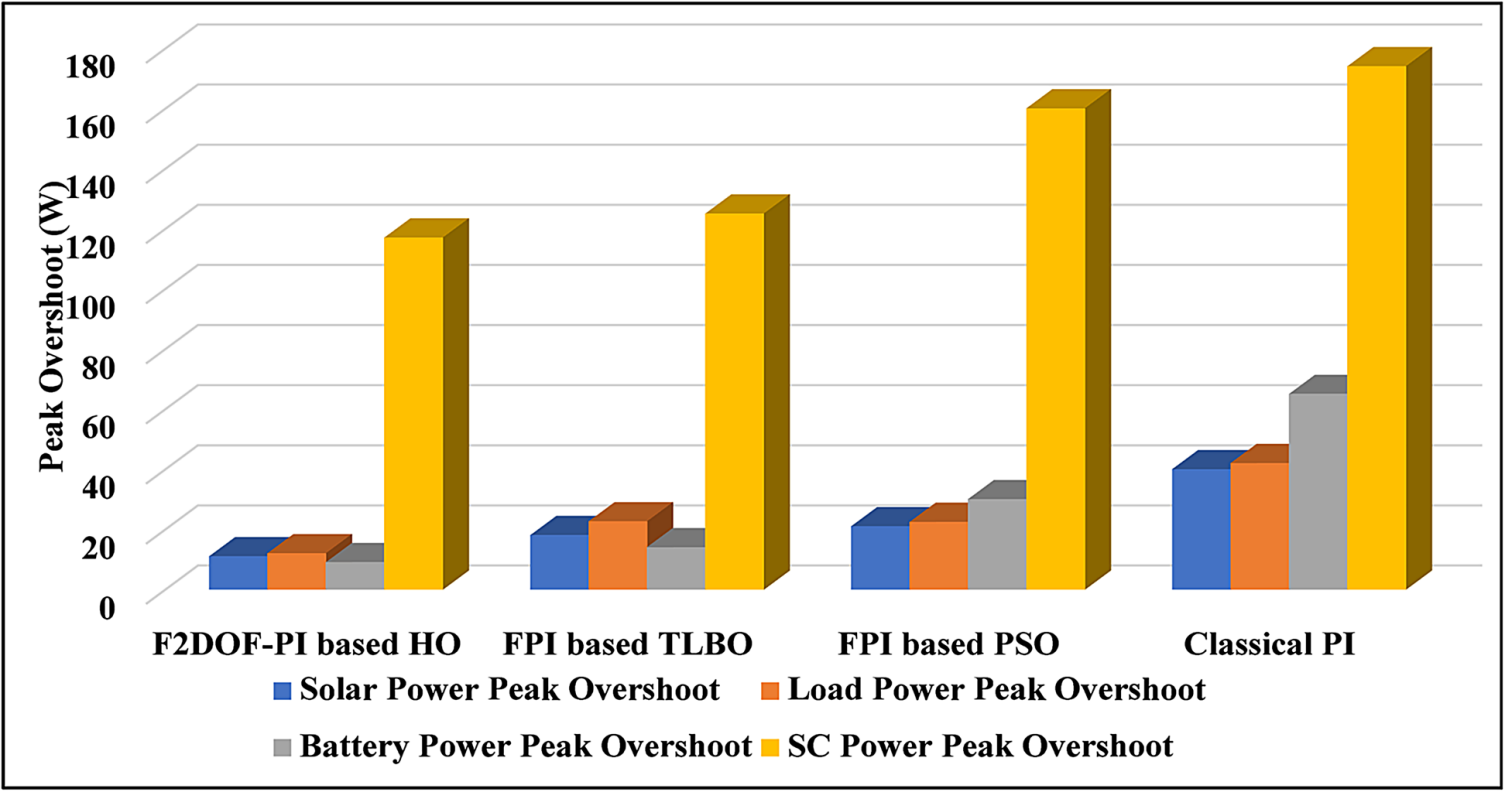
Peak overshoot for different controllers.

**Fig. 26:**
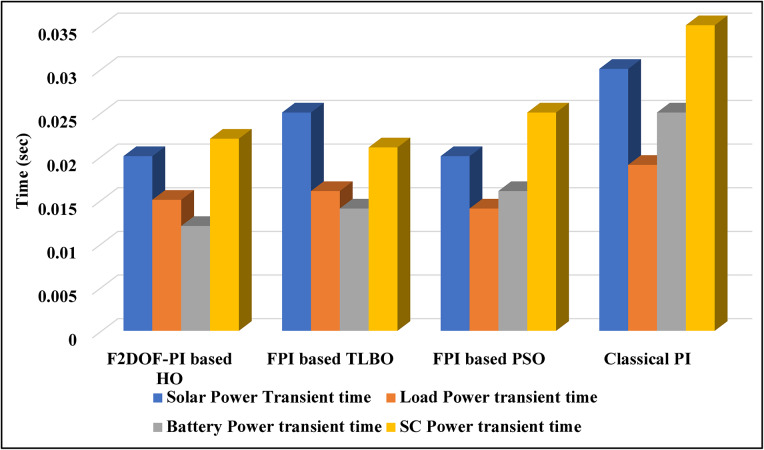
Transient time for different controllers.

To assess the system’s dynamic response and load-sharing efficiency, a step load increase is implemented in this scenario. First, the (HESS), which includes a battery, and the photovoltaic (PV) array work together to keep the overall system load constant. A realistic scenario, like turning on an extra appliance or piece of equipment, is represented by a sudden step increase in load demand that happens at a particular point in the simulation. The solar array provides a significant amount of power before the load increases, with the battery making up the difference. The battery can lower its discharge rate or even recharge if there is excess solar energy available as the PV system gradually takes on more of the load burden as it adapts to the new load condition, possibly using maximum power point tracking (MPPT) mechanisms. Figures 22 and 23 depict the comparative analysis of power responses for various control strategies. Figure 24 illustrates the battery SoC. The peak overshoot and transient time for the various controllers are illustrated in Figs. 25 and 26, respectively. The presenented outcomes reveals that the Fuzzy-2DOF-PI based HO delivers the best performance in terms of both stability and dynamic behavior. While all methods keep the SoC close to 50%, the Fuzzy-2DOF-PI based HO exhibits the smallest deviation, enhancing overall stability. In terms of peak overshoot, the highest supercapacitor (SC) power overshoot is observed in the Classical PI at about 175 W, followed by FPI based PSO (165 W), FPI based TLBO (135 W), and the lowest in F2DOF-PI based HO (125 W). Likewise, battery power overshoot is greatly minimized with F2DOF-PI based HO (15 W) compared to the Classical PI (70 W). For transient performance, the SC power transient time drops from 0.036 s in Classical PI to 0.023 s in F2DOF-PI based HO, while the battery power transient time decreases from 0.028 s to 0.013 s. Overall, the results demonstrate that Fuzzy-2DOF-PI based HO achieves faster settling, lower overshoot, and improved stability over conventional and other optimized PI-based techniques.

### Scenario 3: step load decrease

**Fig. 27:**
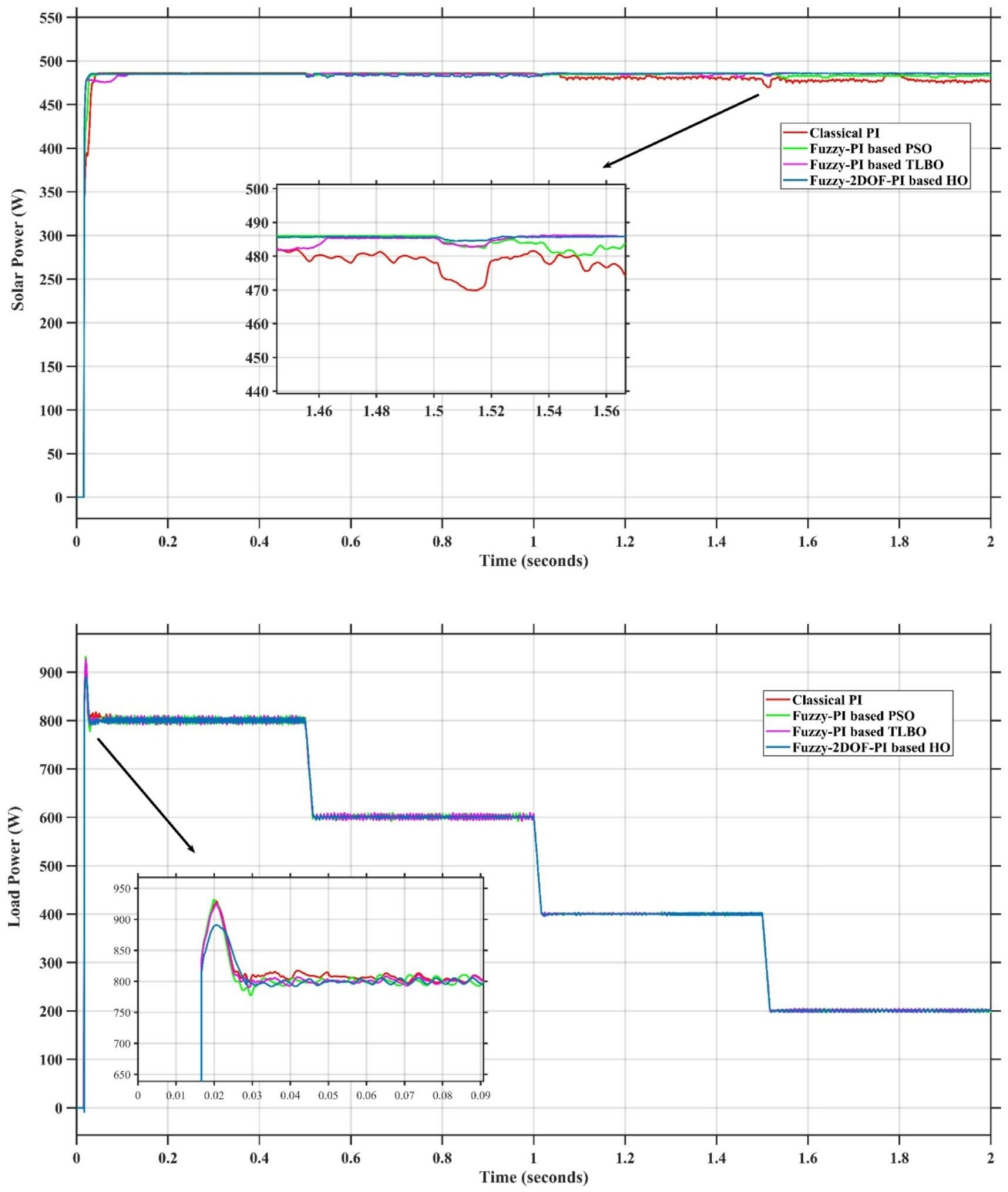
Responses of Solar and Load powers for different controllers.

**Fig. 28:**
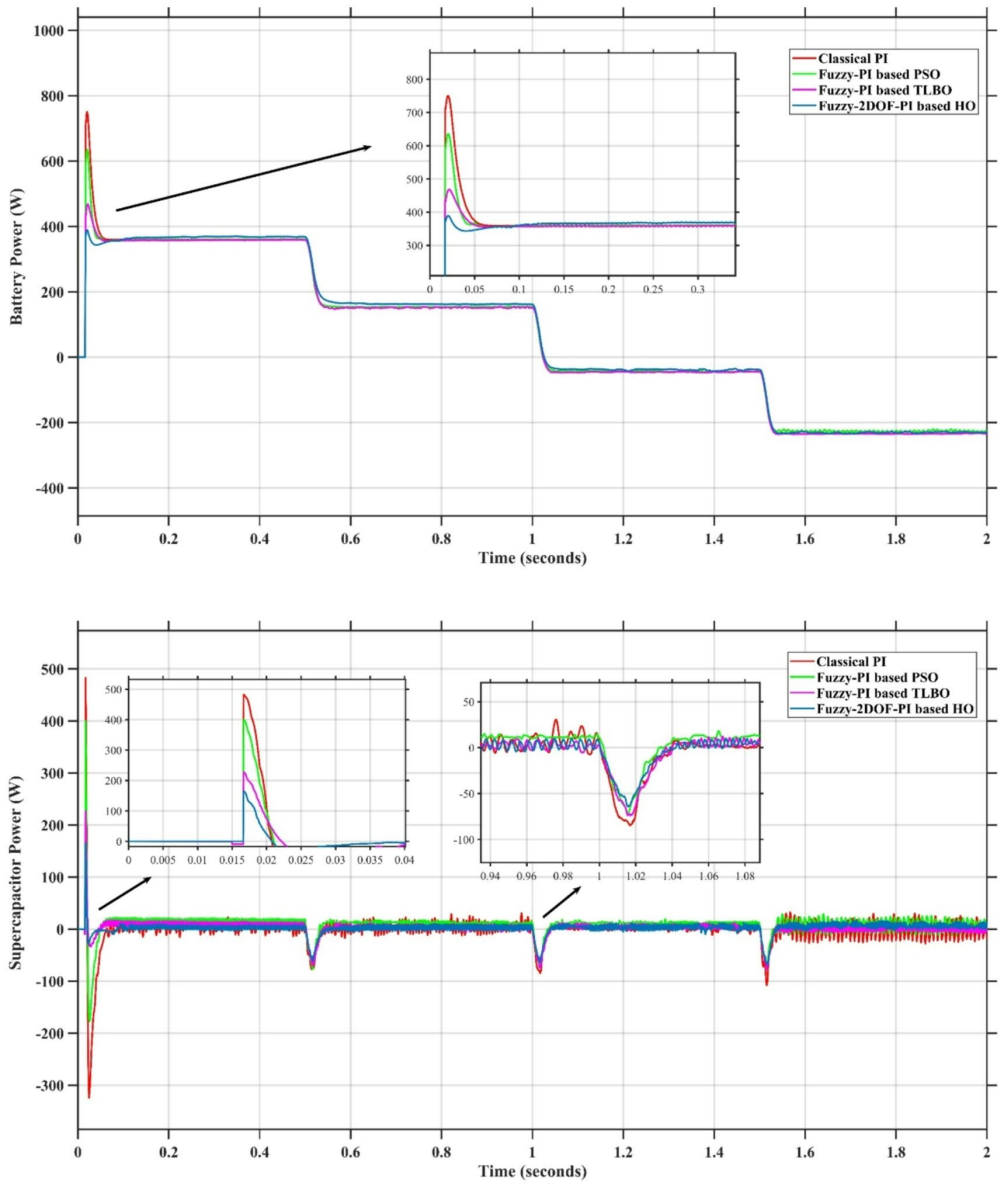
Responses of Battery and Supercapacitor Responses for different controllers.

**Fig. 29:**
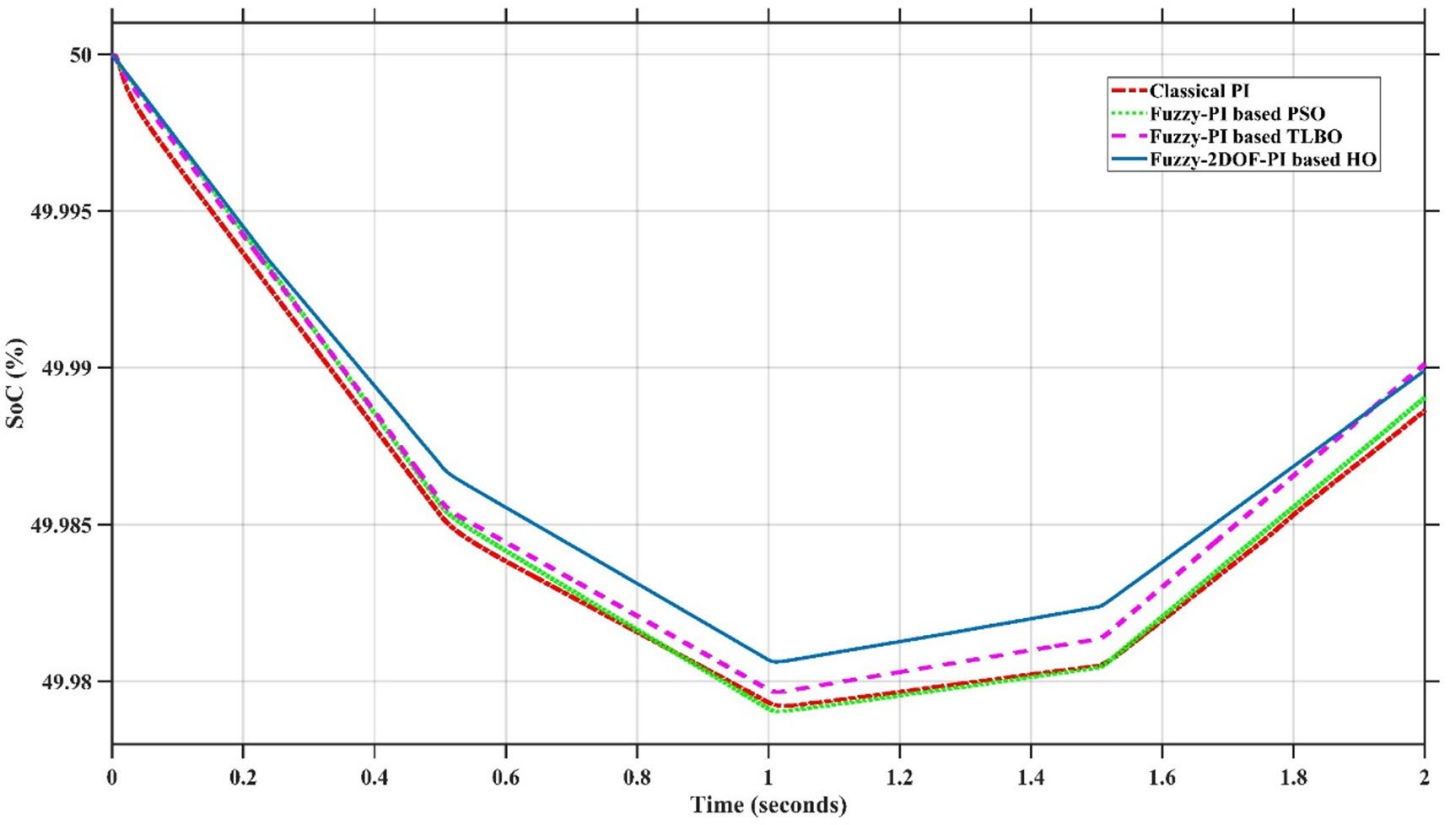
Battery State of Charge.

**Fig. 30:**
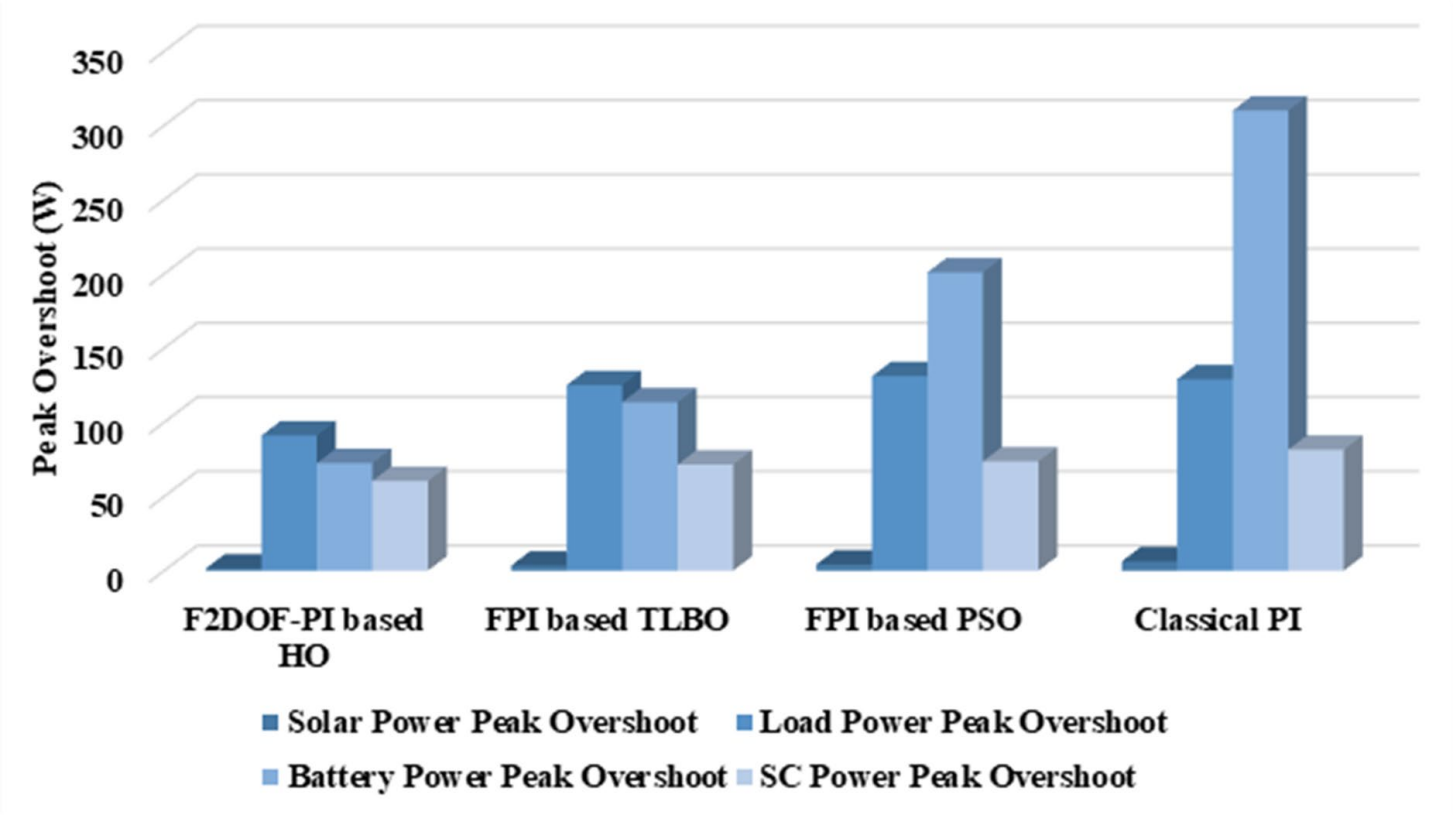
Peak overshoot for different controllers.

**Fig. 31:**
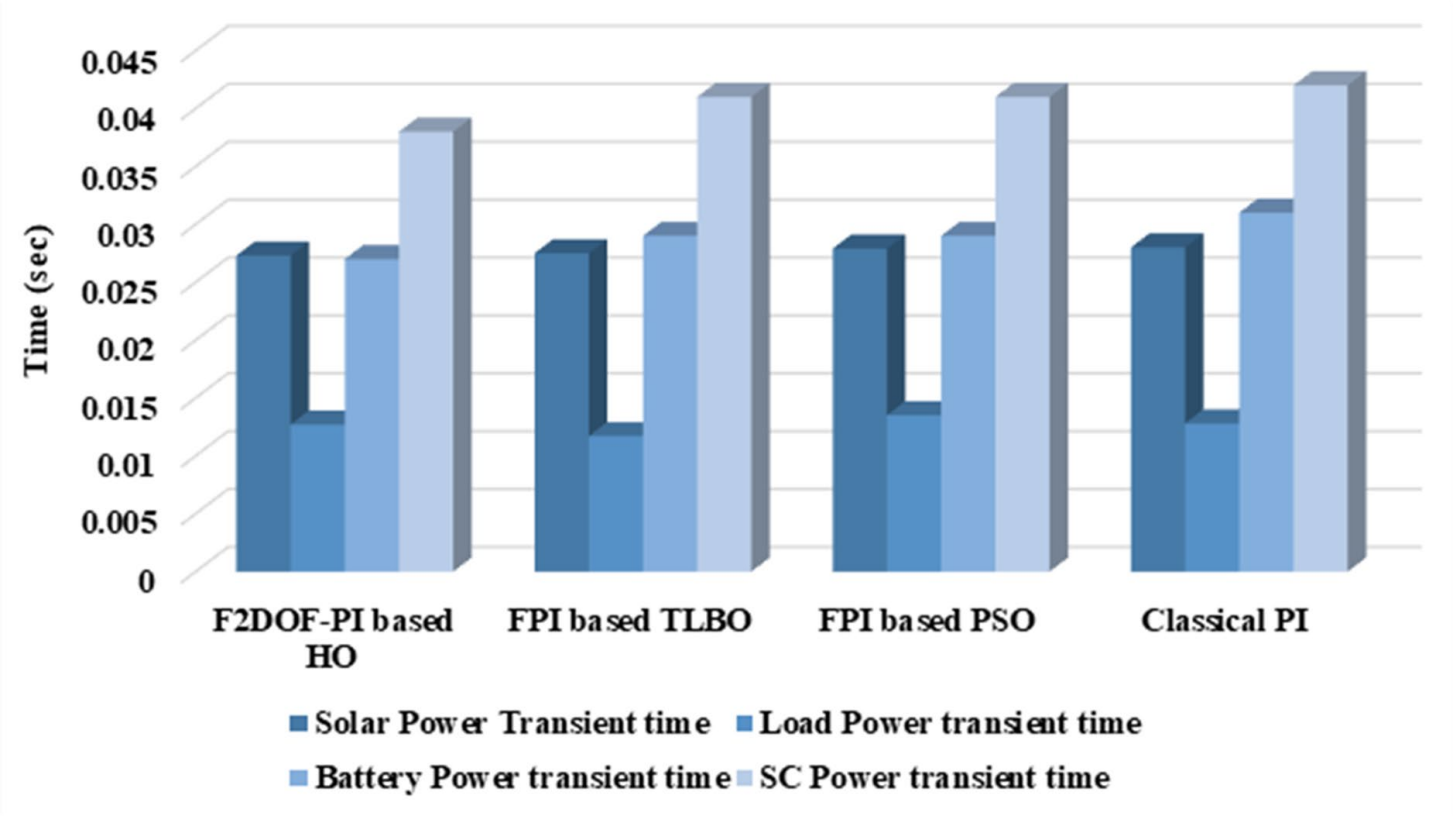
Transient time for different controllers.

In this scenario, a step load decrease is introduced to assess the system’s dynamic response and the effectiveness of power redistribution between the photovoltaic (PV) system and HESS. Initially, the total system load is stable, and power is jointly supplied by the PV array and the battery. At a defined moment during the simulation, the load demand experiences a sudden drop, simulating a real-world event such as the disconnection of a heavy appliance or reduction in operational demand. Prior to the load reduction, the battery supports the solar array by supplying the necessary deficit to maintain load power. However, following the step decrease, the total load demand falls below the available solar generation. As a result, the battery transitions from discharging to charging mode, effectively absorbing the excess power produced by the PV array. Figures 27 and 28 depict the comparative analysis of power responses for various control strategies. Figure 29 illustrates the battery SoC, indicating the periods of charging and discharging in relation to load demand and available solar irradiation. The peak overshoot and transient time for the various controllers are illustrated in Figs. 30 and 31, respectively. The presented results demonstrate that the proposed F2DOF-PI based HO consistently outperforms the others in terms of State of Charge (SoC) regulation, peak overshoot minimization, and transient performance. As shown in the SoC response, all controllers maintain values close to 50%, yet the HO-based method exhibits smaller dips during transient phases and faster recovery compared to the slower Classical PI. In peak overshoot evaluation, the HO approach achieves the lowest values across solar, battery, load, and supercapacitor (SC) power, with significant reductions in load power peaks compared to the excessive overshoot observed in the Classical PI. For battery and SC power regulation, HO further minimizes stress on energy storage components, enhancing system reliability. In terms of transient time, all methods maintain solar power settling near 0.03 s; however, HO achieves the shortest load power transient (about 0.015 s) and faster SC stabilization (near 0.04 s), confirming its good dynamic adaptability.

### Scenario 4: variation of load penetration and solar irradiance

**Fig. 32:**
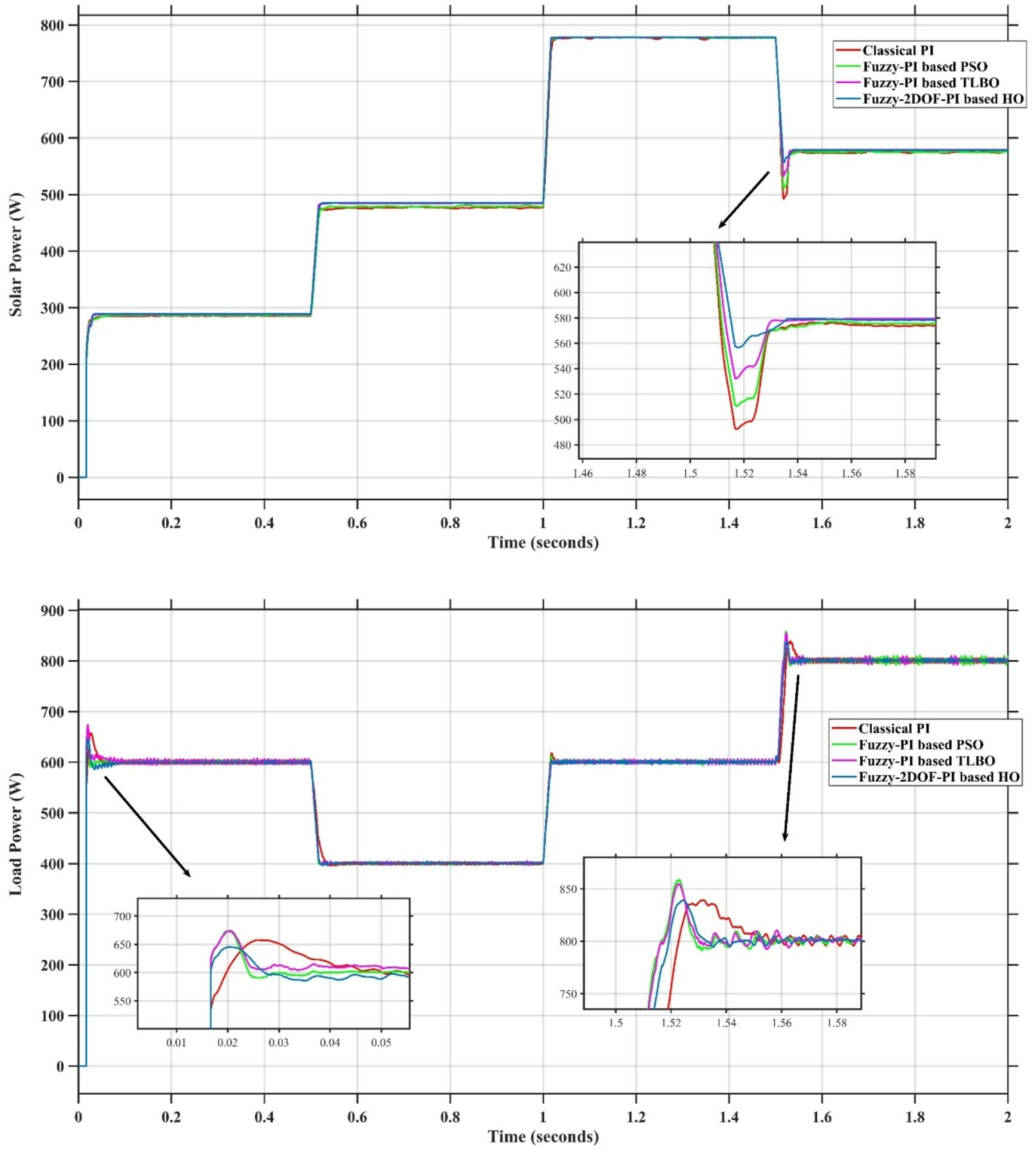
Responses of Solar and Load powers for different controllers.

**Fig. 33:**
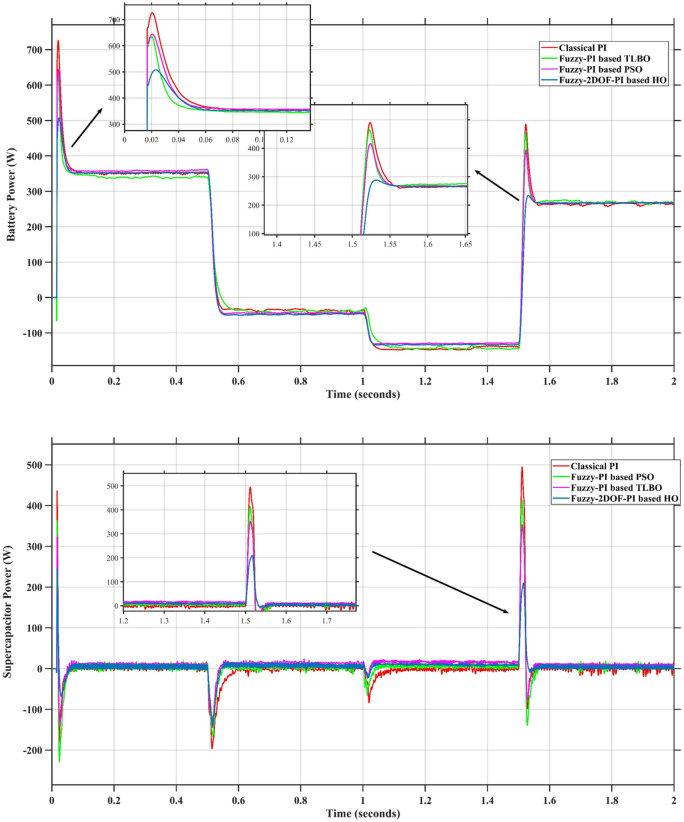
Responses of Battery and Supercapacitor Responses for different controllers.

**Fig. 34:**
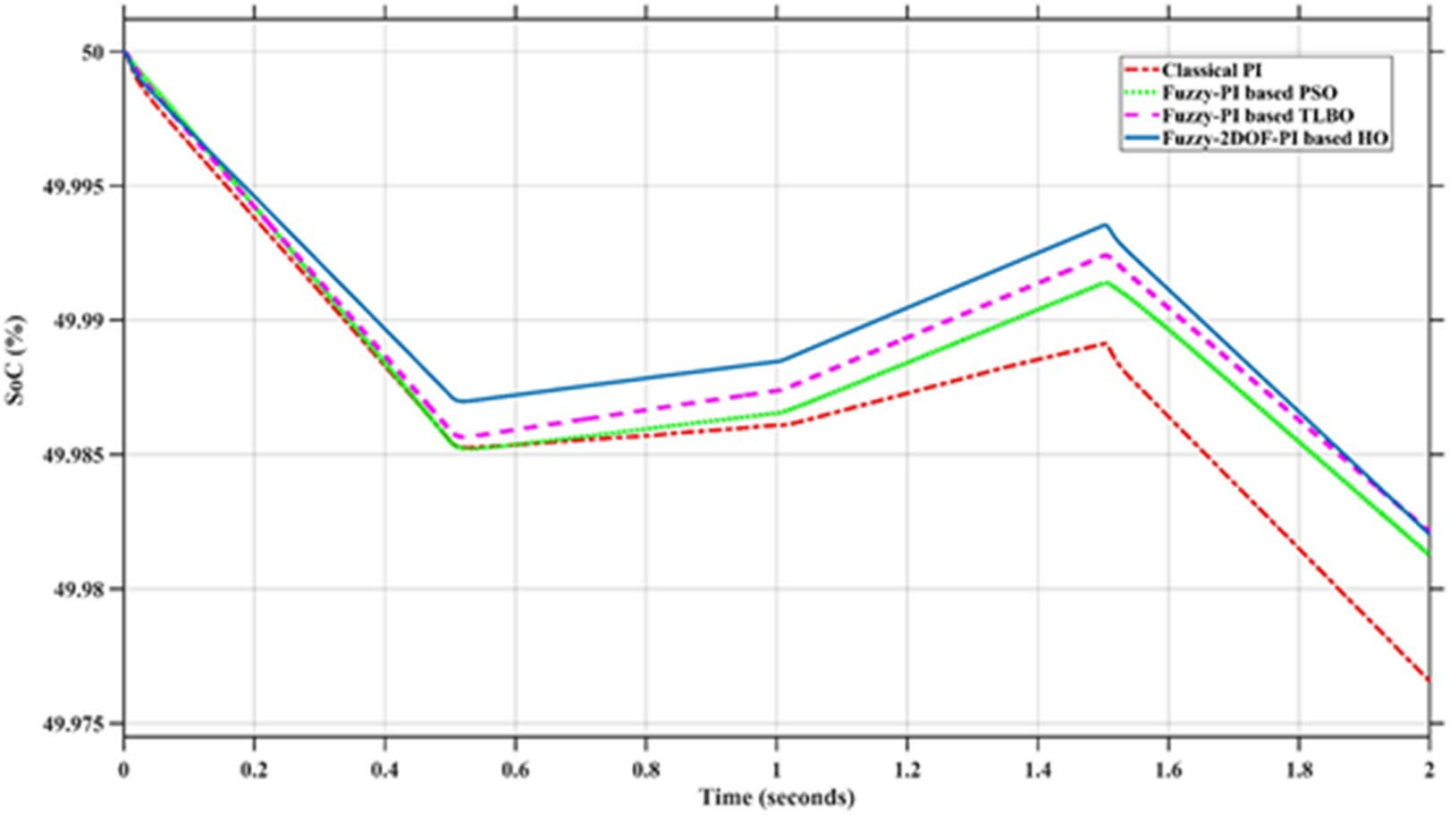
Battery State of Charge.

**Fig. 35:**
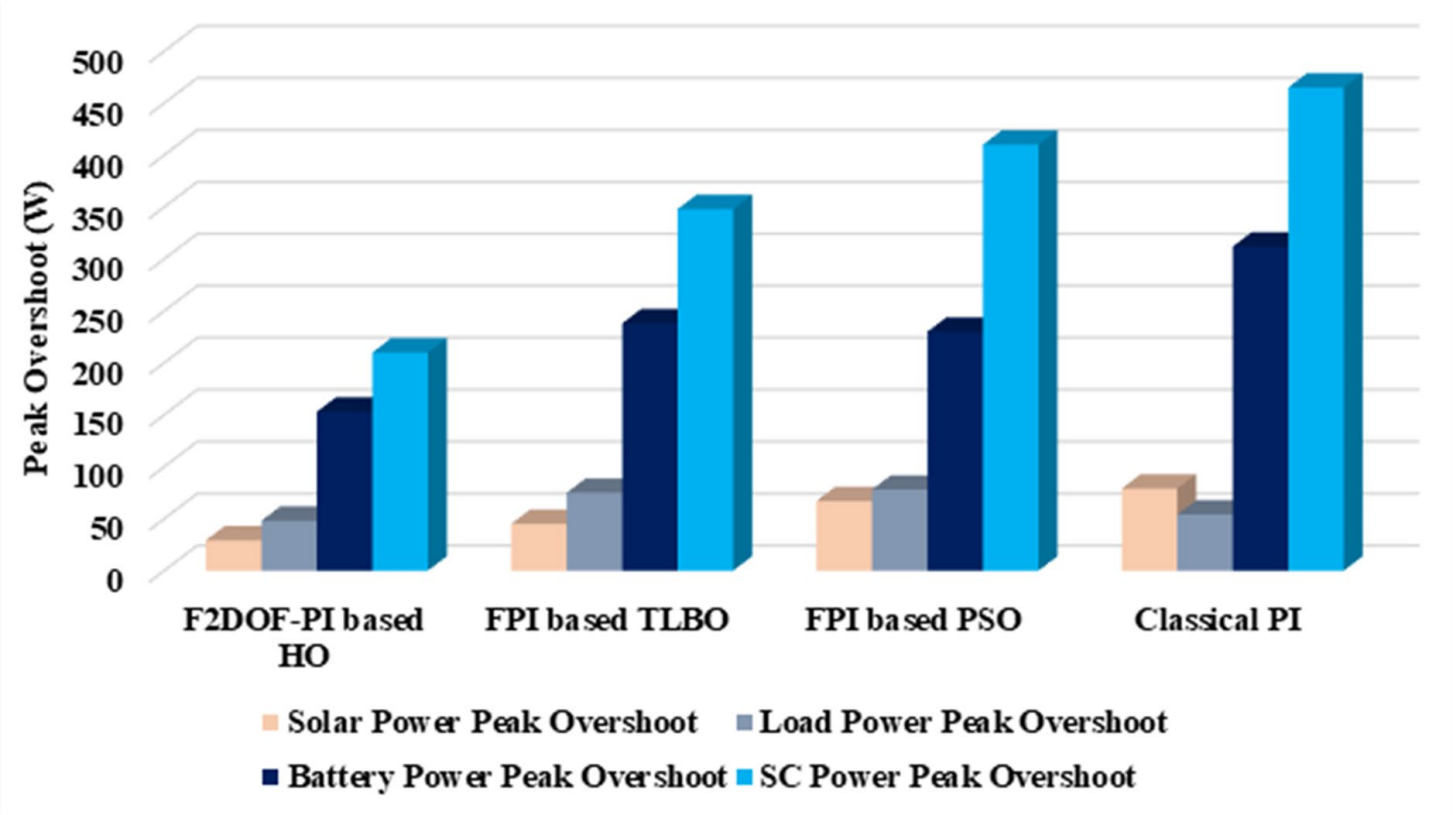
Peak overshoot for different controllers.

**Fig. 36:**
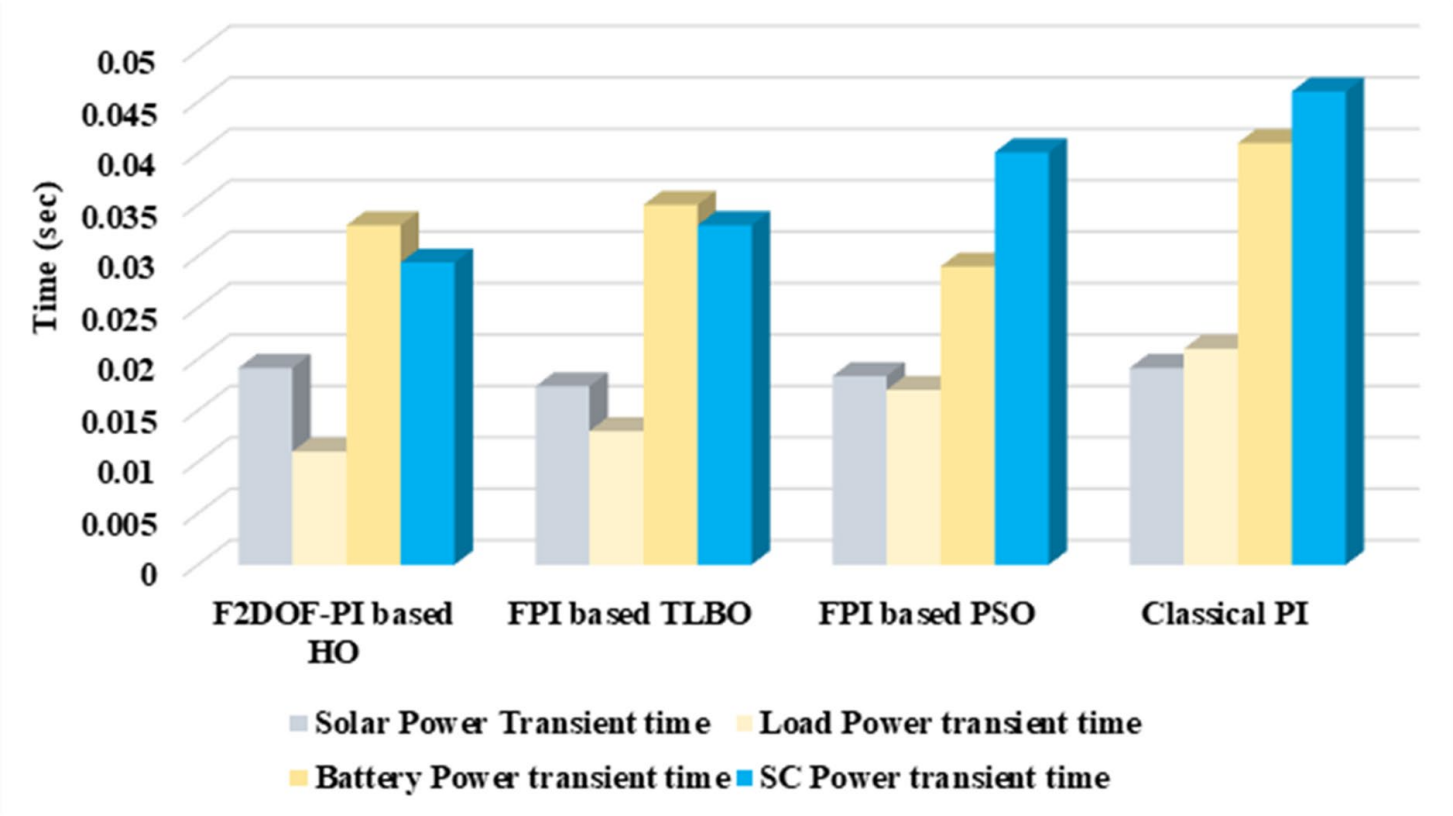
Transient time for different controllers.

In this scenario, the system is subjected to simultaneous variations in both load penetration and solar irradiance to evaluate the robustness and adaptability of the control strategies under more complex and realistic operating conditions. This mixed disturbance scenario mimics practical situations such as fluctuating consumer demand coupled with intermittent solar energy availability due to passing clouds or weather changes. Initially, the PV system and battery within HESS operate together to meet stable demand. As the simulation progresses, both a step change in solar irradiance and a variation in load demand are introduced. These concurrent changes challenge the system’s ability to maintain power balance and ensure uninterrupted load supply. The battery plays a critical compensatory role, dynamically shifting between charging and discharging modes in response to the net power imbalance resulting from fluctuating solar input and load variations. Figures 32 and 33 present the comparative analysis of power responses under various control techniques, while Fig. 34 illustrates the battery’s state of charge, showcasing its smooth behavior during simultaneous changes. The system’s transient response and peak overshoot under these compounded conditions are depicted in Figs. 35 and 36, respectively. The analysis of both transient time and peak overshoot results highlights the superior performance of the F2DOF-PI based HO controller. In terms of transient time, it achieves fast responses of approximately 0.021 s for solar power and 0.035 s for battery power, outperforming all other controllers. The classical PI, on the other hand, shows significantly slower responses, with 0.023 s for load power and 0.049 s for supercapacitor power, indicating delayed system settling. For peak overshoot, the proposed method records notably low values, such as 40 W for solar power and 230 W for supercapacitor power, reflecting reduced transient stress. By contrast, the classical PI reaches overshoots of 95 W and 490 W in the same categories, which can accelerate component degradation. The observed differences confirm that the proposed approach improves both dynamic stability and steady-state accuracy in PV-HESS control. Compared with optimization-based FPI controllers, the F2DOF-PI based HO achieves a better trade-off between response time and overshoot minimization.

### Steady state error analysis

**Table 6:** Percentage steady-state error of $\:{P}_{\mathrm{solar}}$, $\:{P}_{\mathrm{load}}$, and $\:{P}_{B}$ for different controllers under four operating scenarios.

Controller	Percentage steady state error of *P*_Solar_	Percentage steady state error of *P*_load_	Percentage steady state error of *P*_B_
	Scenario 1		
Conventional PI	1.81	0.52	1.54
FPI- based PSO	1.76	0.47	1.42
FPI-based TLBO	0.37	0.31	1.12
F-2DOFPI-based HO (Proposed)	0.21	0.25	1.05
	*Scenario 2*		
Conventional PI	2.13	0.77	1.75
FPI- based PSO	2.07	0.74	1.82
FPI-based TLBO	1.95	0.65	1.05
F-2DOFPI-based HO (Proposed)	1.86	0.59	0.96
	*Scenario 3*		
Conventional PI	0.54	1.08	1.55
FPI- based PSO	0.42	1.05	1.57
FPI-based TLBO	0.28	0.83	1.35
F-2DOFPI-based HO (Proposed)	0.21	0.51	1.33
	*Scenario 4*		
Conventional PI	0.25	0.6	1.97
FPI- based PSO	0.21	0.58	2.04
FPI-based TLBO	0.19	0.48	1.84
F-2DOFPI-based HO (Proposed)	0.12	0.33	1.39

Table 6 presents a quantitative comparison of the percentage steady-state errors of solar power $\:{P}_{\mathrm{solar}}$, load power $\:{P}_{\mathrm{load}}$, and battery power $\:{P}_{B}$under four operating scenarios for all investigated controllers. The results clearly demonstrate that the proposed F-2DOFPI-based HO controller consistently achieves the lowest steady-state errors across all scenarios and power components. In Scenario 1, the proposed method reduces the steady-state error of $\:{P}_{\mathrm{solar}}$to 0.21%, compared with 1.81% for the conventional PI and 0.37% for the TLBO-based fuzzy PI. Similar trends are observed in Scenarios 2–4, where the proposed controller maintains smaller deviations in both $\:{P}_{\mathrm{load}}$and $\:{P}_{B}$, indicating improved power tracking accuracy and more effective energy sharing within the hybrid energy storage system. Overall, the results confirm that integrating a 2DOF-PI structure with fuzzy supervision and HO-based optimization significantly enhances steady-state performance and robustness compared to classical and other optimization-based fuzzy PI controllers.

### Stability and ablation analysis

The stability performance of the examined controllers is evaluated under progressive load increase scenarios of 60%, 65%, 68%, 71%, and 73%, as shown in Table 7. With 60% load increase, all controllers continue to operate steadily, demonstrating nominal performance under moderate loading circumstances. However, all optimized fuzzy based controllers maintain stable operation when the load reaches 65%, demonstrating the efficacy of intelligent tuning strategies in enhancing disturbance rejection capability. In contrast, the conventional PI controller is unable to maintain system stability at this point. While the FPI-based TLBO and the F-2DOFPI based HO controllers continue to maintain stable system behavior, the FPI-based PSO controller becomes unstable at a 68% load increase. This outcome shows that TLBO and HO optimization techniques are more robust than PSO-based tuning. The suggested F-2DOFPI-based HO controller is the only one that maintains stability when the load increase exceeds 71%, demonstrating its capacity to improve system stability margins under extreme loading circumstances. Finally, all controllers lose stability at a 73% load increase, revealing the system’s operational stability limit under the control techniques under consideration. When compared to traditional PI, FPI-PSO, and FPI-TLBO controllers, the comparison study clearly shows that the suggested F-2DOFPI-based HO controller greatly expands the stability region and offers improved robustness against major load perturbations.

**Table 7:** Evaluation of control system robustness against load increase.

Controller	Load Increase (%)				
	60%	65%	68%	71%	73%
Conventional PI	stable	instable	instable	instable	instable
FPI- based PSO	stable	stable	instable	instable	instable
FPI-based TLBO	stable	stable	stable	instable	instable
F-2DOFPI-based HO (Proposed)	stable	stable	stable	stable	instable

To further evaluate the contribution of the optimization technique to controller performance, an ablation study was conducted by comparing the conventional 2DOF-PI controller with the optimized F-2DOF-PI controller based on HO. The optimized F-2DOF-PI-based HO achieved a lower objective function value (5307.7) compared with the conventional 2DOF-PI controller (5687.4), indicating improved control performance. This performance enhancement confirms the effectiveness of the optimization process in refining controller parameters. Therefore, the ablation analysis highlights the positive impact of integrating HO optimization within the F-2DOF-PI control structure compared with the non-optimized counterpart.

## Conclusion and outlook

This study investigated the design, control, and performance evaluation of a photovoltaic (PV) system integrated with a parallel active hybrid energy storage system (HESS) composed of a battery pack and a supercapacitor. The HESS was shown to play a critical role in maintaining DC-link voltage stability and balancing power generation and demand. To enhance system performance, an advanced control strategy combining fuzzy logic with a two-degree-of-freedom PI (2DOF-PI) controller, optimally tuned using the Hippopotamus Optimization (HO) algorithm, was proposed. Acting as the main regulator, the proposed fuzzy 2DOF-PI controller ensured stable bidirectional power exchange through DC–DC converters and effective DC bus voltage regulation with reduced computational complexity under fluctuating operating conditions. Simulation results demonstrated that the proposed control scheme effectively maintains reliable operation during sudden variations in solar irradiance and load demand. The battery was responsible for supplying the steady-state power component, while the supercapacitor absorbed fast transient fluctuations, enabling efficient power sharing within the HESS. Moreover, the control strategy ensured appropriate battery charging and discharging behavior, with the supercapacitor mitigating high-frequency disturbances and supporting stable, uninterrupted power delivery to the load. Overall, the results confirm that integrating a fuzzy 2DOF structure with HO-based optimization yields better power regulation performance compared to conventional and other optimized PI-based controllers. Despite the encouraging simulation results, the proposed approach has not yet been validated through experimental or hardware-in-the-loop testing, and the component aging were not explicitly considered. Future work will address real-time implementation and comprehensive robustness evaluation under practical operating conditions, with emphasis on uncertainty-aware and adaptive control enhancements. In particular, the influence of State of Charge (SOC) estimation errors for both the battery and supercapacitor will be investigated. Observer-based SOC estimation techniques and sensitivity analyses will be incorporated to assess their effects on power-sharing accuracy, protection constraint enforcement, and overall system stability.

## Data Availability

The datasets used and/or analysed during the current study available from the corresponding author on reasonable request.
